# Development and Interpretability Analysis of a Stacking Ensemble Model for Early Prediction of Nutritional Risk in Intensive Care Unit Patients: Retrospective Cohort Study

**DOI:** 10.2196/77872

**Published:** 2026-06-03

**Authors:** Xu Zhang, An Fang, Pei Lou, Kuanda Yao, Tianci Huang, Jiahui Hu

**Affiliations:** 1 Institute of Medical Information/Medical Library, Chinese Academy of Medical Sciences and Peking Union Medical College, No. 3 Yabao Road, Chaoyang District, Beijing, 100020, China, 86 01052328782; 2 State Key Laboratory of Networking and Switching Technology, Beijing University of Posts and Telecommunications, Beijing, China

**Keywords:** malnutrition, intensive care units, machine learning, stacking ensemble, model interpretability

## Abstract

**Background:**

Malnutrition in critically ill patients is associated with increased morbidity and mortality, yet traditional screening tools such as the modified NUTRIC (mNUTRIC) score often rely on subjective assessments or delayed data, limiting their utility for early intervention in the dynamic intensive care unit (ICU) environment. Real-time, data-driven approaches using electronic health records offer a promising solution for automated and objective risk stratification.

**Objective:**

This study aimed to develop and validate a machine learning model, the E-NUTRIC (Ensemble-NUTRIC), for the early prediction of malnutrition risk within the first 24 hours of ICU admission. By integrating multiple algorithms through stacking ensemble learning, we sought to improve predictive performance over traditional scoring systems and individual machine learning models while maintaining clinical interpretability.

**Methods:**

We conducted a retrospective cohort study using data from the Medical Information Mart for Intensive Care (MIMIC-IV, version 3.1). Adult ICU stays exceeding 24 hours were included, and the primary outcome was malnutrition diagnosis. Variables from the first 24 hours (demographics, vitals, and laboratory tests) were extracted and harmonized. Missingness was addressed with k-nearest neighbors imputation, features were standardized, and class imbalance was mitigated via random undersampling. The proposed E-NUTRIC model used a stacking ensemble with 4 base learners—Logistic Regression, Random Forest, Extreme Gradient Boosting (XGBoost), and Light Gradient Boosting Machine—and a logistic metalearner. Performance was assessed on a stratified 80/20 holdout test set using area under the receiver operating characteristic curve (AUROC), area under the precision-recall curve, and calibration curves. The mNUTRIC score served as the clinical benchmark. Model interpretability was derived by applying Shapley Additive Explanations (SHAP) specifically to the highly predictive XGBoost component, while clinical utility was assessed using Platt scaling recalibration.

**Results:**

The final cohort comprised 51,483 patients, of whom 4384 (8.5%) were classified as being at malnutrition risk. The E-NUTRIC model showed superior discrimination with an AUROC of 0.875 (95% CI 0.864‐0.885), outperforming the mNUTRIC score (AUROC=0.635, *P*<.001). Relative to individual base learners, E-NUTRIC achieved the best overall performance, exceeding those of the best-performing individual models XGBoost (AUROC=0.871) and Light Gradient Boosting Machine (AUROC=0.866). The area under the precision-recall curve of E-NUTRIC was 0.424, representing approximately a 3.4-fold increase over mNUTRIC (0.126). SHAP analysis highlighted minimum serum albumin, admission weight, early hypokalemia, and specific ICU admission types as key nonlinear predictors of malnutrition risk. Unlike the traditional mNUTRIC score, which compressed predictions into a low-risk tier, the recalibrated E-NUTRIC model effectively spanned the full probability spectrum, thereby isolating high-risk phenotypes.

**Conclusions:**

The E-NUTRIC stacking ensemble provides an interpretable approach for nutritional risk screening in the ICU using routinely available electronic health records data. In this retrospective cohort study, it demonstrated superior discrimination to the mNUTRIC score and offered clinically consistent feature attributions.

## Introduction

Malnutrition is a critical prognostic risk factor for patients in the intensive care unit (ICU), significantly influencing clinical outcomes such as length of ICU stay, duration of mechanical ventilation, infection risk, and mortality [1-3]. A meta-analysis of 20 studies reported that the prevalence of malnutrition among ICU patients ranges from 38% to 78%. The physiological stress of critical illness exacerbates this vulnerability, with nutritional risk escalating during prolonged hospitalization [4]. Crucially, research indicates that early prediction and identification of malnourished patients can facilitate timely and appropriate nutritional interventions, thereby mitigating these adverse outcomes. A national cohort study in Switzerland demonstrated that nutritional support significantly reduced patients’ all-cause mortality, reduced 30-day rehospitalization rates, and increased the likelihood of discharge to home [5].

Clinical guidelines recommend that critically ill patients undergo nutritional risk screening within 48 hours of ICU admission to guide therapy [6]. However, the widely used nutritional risk screening tools, such as the Nutritional Risk Screening 2002 [7] and the modified NUTRIC score (mNUTRIC) [8], along with biomarkers such as plasma albumin, have recognized limitations in the ICU setting. These methods can be subjective, require manual data collection which is often delayed, and have demonstrated low recommendation levels for accurately assessing and predicting nutritional status in this dynamic population [9,10]. This highlights a clear need for an objective and early detection method for malnutrition risk in the ICU.

To meet this need, researchers have begun to explore machine learning (ML) algorithms. Early studies often used traditional ML models such as Extreme Gradient Boosting (XGBoost) and Logistic Regression (LR), demonstrating the potential of using electronic health records (EHRs) data to predict malnutrition [11,12]. However, single model approaches often struggle with generalization across diverse patient populations and may suffer from overfitting on imbalanced datasets [13]. Furthermore, previous studies frequently used data from the entire hospitalization period, introducing “look-ahead bias” that limits their utility for early prospective screening [14]. Crucially, complex “black-box” models often lack interpretability, hindering clinical adoption as clinicians require transparent reasoning behind risk predictions to make informed decisions [15]. Few studies have rigorously benchmarked advanced ensemble methods against established clinical scoring systems such as mNUTRIC within a strictly prospective early prediction framework.

This study uses the Medical Information Mart for Intensive Care (MIMIC-IV) database (v3.1) [16], a large, publicly available repository containing comprehensive clinical data. This rich dataset enables the development of robust predictive models based on routinely collected parameters. However, analyzing real-world EHR data presents significant challenges, including high rates of missing data, heterogeneity in variable recording, and pronounced class imbalance, as malnutrition is less common than normal nutritional status [17]. Addressing these challenges through rigorous data preprocessing and advanced modeling strategies is essential for building a reliable clinical decision support tool.

Therefore, the objective of this study is to develop and validate an interpretable stacking ensemble model, named E-NUTRIC (Ensemble-NUTRIC), for the early prediction of malnutrition risk in ICU patients using data exclusively from the first 24 hours of admission. We propose a Stacking Ensemble Learning framework that integrates multiple base learners, including LR, Random Forest (RF), XGBoost, and Light Gradient Boosting Machine (LightGBM), to enhance predictive stability and accuracy compared with individual algorithms. By integrating a well-established ML interpretability framework, Shapley Additive Explanations (SHAP) [18,19], we aim to enhance the transparency and clinical applicability of our model. Ultimately, this work seeks to provide a precise and automated screening tool that outperforms traditional scoring systems, enabling clinicians to intervene earlier and improve patient outcomes.

## Methods

### Data Source and Study Population

This study was a retrospective cohort analysis using the MIMIC-IV database (version 3.1) [16]. MIMIC-IV is a large, single-center, publicly available relational database containing comprehensive clinical data from patients admitted to the Beth Israel Deaconess Medical Center in Boston, Massachusetts, between 2008 and 2019. The database was approved by the Institutional Review Boards of the Massachusetts Institute of Technology and Beth Israel Deaconess Medical Center. The requirement for individual patient informed consent was waived because all protected health information was deidentified.

**Figure 1.**
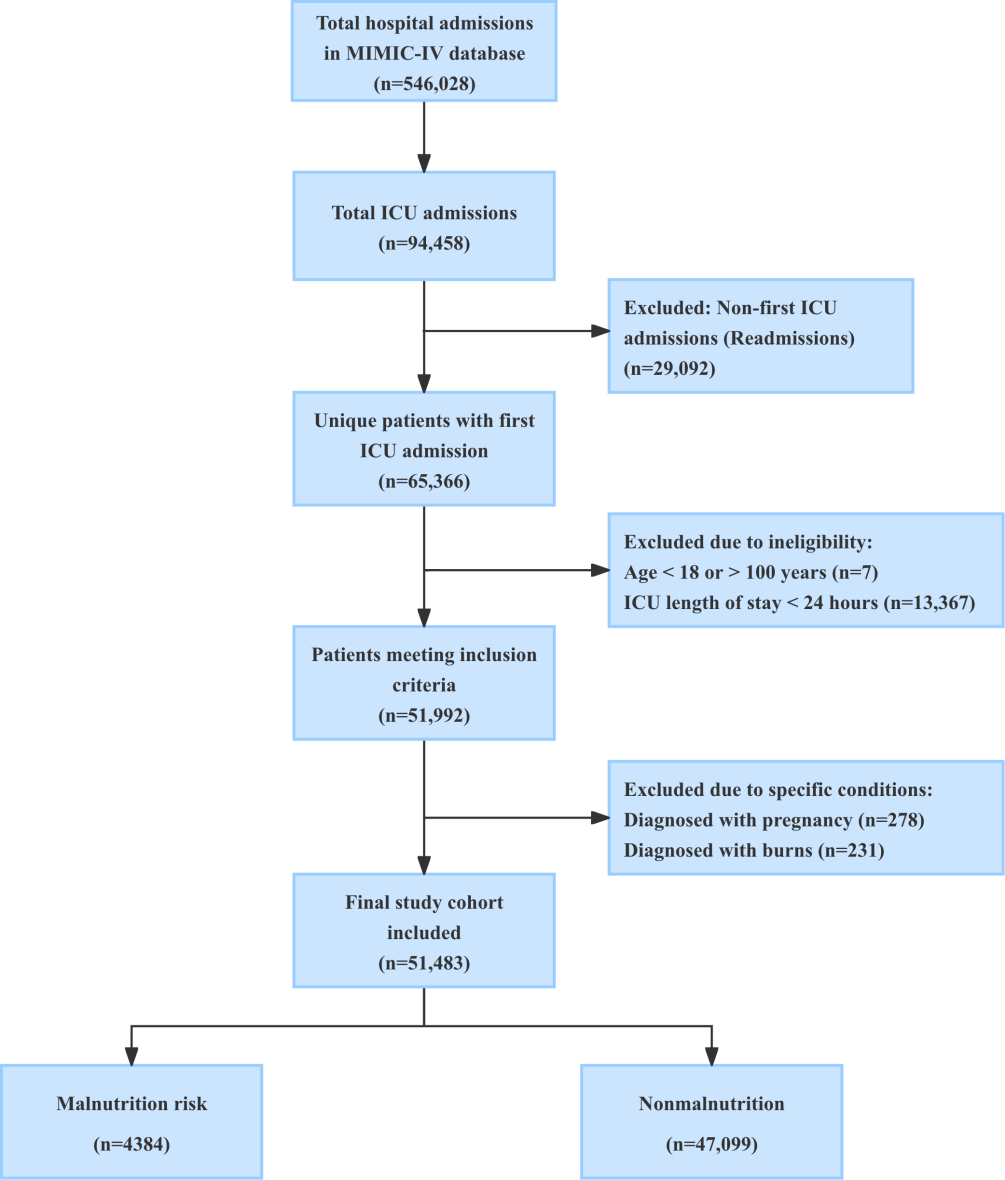
Flowchart of patient selection. The diagram illustrates the inclusion and exclusion process applied to the MIMIC-IV database. Starting from total hospital admissions, we sequentially filtered for ICU patients, first admissions, valid age ranges, and sufficient length of stay. Patients with confounding conditions (pregnancy and severe burns) were excluded. The final cohort (n=51,483) was stratified into the malnutrition risk group (defined by *ICD-9* [*International Classification of Diseases, Ninth Revision*] and *ICD-10* [*International Statistical Classification of Diseases, Tenth Revision*] codes) and the nonmalnutrition group for model development. ICU: intensive care unit; MIMIC-IV: Medical Information Mart for Intensive Care.

We systematically screened all hospital admissions in the MIMIC-IV database to identify eligible patients. To ensure data consistency and minimize bias from repeated measures, we restricted our analysis to the first ICU admission of the first hospitalization for each patient. Patients younger than 18 years or older than 100 years, as well as those with an ICU stay of <24 hours, were excluded from both the positive and negative cohorts. Figure 1 presents the data flow diagram.

For the positive cohort (patients with malnutrition), we initially identified 23,099 hospital admissions with a diagnosis of malnutrition. After applying these malnutrition diagnosis criteria and excluding non–first hospitalizations and non–first ICU stays, 5081 patients remained. Further applying the age and ICU stay exclusions yielded a final positive cohort of 4384 patients.

For the negative cohort (patients with no malnutrition), we started with 546,028 total hospital admissions, corresponding to 94,458 ICU admissions. After excluding duplicate ICU admissions, we retained 65,366 first-time ICU patients. Applying the same age and ICU stay exclusions left 51,992 eligible patients. To ensure a clear control group, we further excluded patients diagnosed with conditions that could confound nutritional status assessment, namely, pregnancy and severe burns. Finally, after removing the 4384 patients already identified in the positive cohort, the final negative cohort comprised 47,099 patients.

The final study population consisted of 51,483 adult patients, with malnutrition prevalence in 4384 (8.5%) patients. The primary outcome was the diagnosis of malnutrition, defined using *ICD-9* (*International Classification of Diseases, Ninth Revision*) codes 260‐263 and *ICD-10* (*International Statistical Classification of Diseases, Tenth Revision*) codes E40–E46. These codes encompass a spectrum of nutritional deficiencies, including kwashiorkor, nutritional marasmus, and severe to mild protein-calorie malnutrition. All data partitioning into training (80%) and test (20%) sets were performed strictly at the patient level using unique participant identifiers to prevent data leakage.

### Feature Extraction and Data Preprocessing

To facilitate early and accurate risk stratification, data extraction was strictly limited to the first 24 hours of ICU admission. This temporal restriction prevents look-ahead bias and ensures that the model relies solely on information available during the early critical phase. We extracted a comprehensive set of clinical variables based on their clinical relevance and data availability in the MIMIC-IV database [16]. These concepts encompassed demographics; vital signs; laboratory tests; therapeutic interventions such as ventilation, dialysis, and vasopressor usage; and clinical severity scores including the Acute Physiology and Chronic Health Evaluation II and the Sequential Organ Failure Assessment (SOFA) scores.

Specific emphasis was placed on laboratory indicators related to nutritional status, inflammation, and metabolic function. The extracted laboratory panel included serum albumin, total cholesterol, lymphocytes, C-reactive protein, erythrocyte sedimentation rate, glucose, lactate, electrolytes, and renal function markers. To capture the dynamic fluctuations inherent in critical illness, we did not rely on single time-point measurements. Instead, for every time-varying vital sign and laboratory parameter, we computed 6 statistical aggregate metrics: mean, SD, minimum, maximum, first value, and last value within the 24-hour window. These statistics, combined with nominal categorical variables, including gender, race, insurance, language, marital status, and ICU unit type, were represented using one-hot encoding with explicit missing-category indicators. After preprocessing and feature expansion, the final model used 296 predictors (see Multimedia Appendix 1 for details).

Before modeling, rigorous data cleaning was performed to address physiological artifacts and measurement errors based on expert consensus [20]. We enforced clinically plausible ranges for key variables; for instance, weight was restricted to 30‐300 kg and BMI to 10‐100 kg/m². Temperature readings required specific handling to harmonize units: values exceeding 50 were interpreted as Fahrenheit and converted to Celsius, after which a valid range of 25 °C to 45 °C was enforced. Values falling outside these predefined thresholds were designated as missing data.

To handle missing data without discarding valuable patient information, we used a k-nearest neighbors (KNN) imputation strategy [21]. This method estimates missing entries by identifying the *k* most similar patients in the feature space, thereby preserving the multivariate correlation structure of the data. We validated this choice through preliminary experiments, where KNN imputation demonstrated superior area under the receiver operating characteristic curve (AUROC) in 5-fold cross-validation compared with simple mean imputation or multivariate imputation by chained equations. Following imputation, all continuous features were standardized using *Z*-score normalization to achieve zero mean and unit variance. This step is critical for algorithms sensitive to feature scaling, such as LR [22], which serves as both a base learner and the metalearner in our ensemble framework.

Given the pronounced class imbalance in our cohort (8.5% malnutrition prevalence), standard training procedures would likely bias the model toward the majority class. To mitigate this, we integrated a random undersampling (RUS) strategy into the training pipeline. Comparative analysis of resampling techniques revealed that RUS yielded a higher area under the precision-recall curve (AUPRC) than Synthetic Minority Over-sampling Technique or class-weighting adjustments. Importantly, this resampling was applied exclusively to the training folds within the cross-validation process, ensuring that the validation and test sets maintained the natural clinical prevalence of malnutrition to provide realistic performance estimates. Preliminary comparisons of imputation methods are detailed in Multimedia Appendix 2.

**Figure 2.**
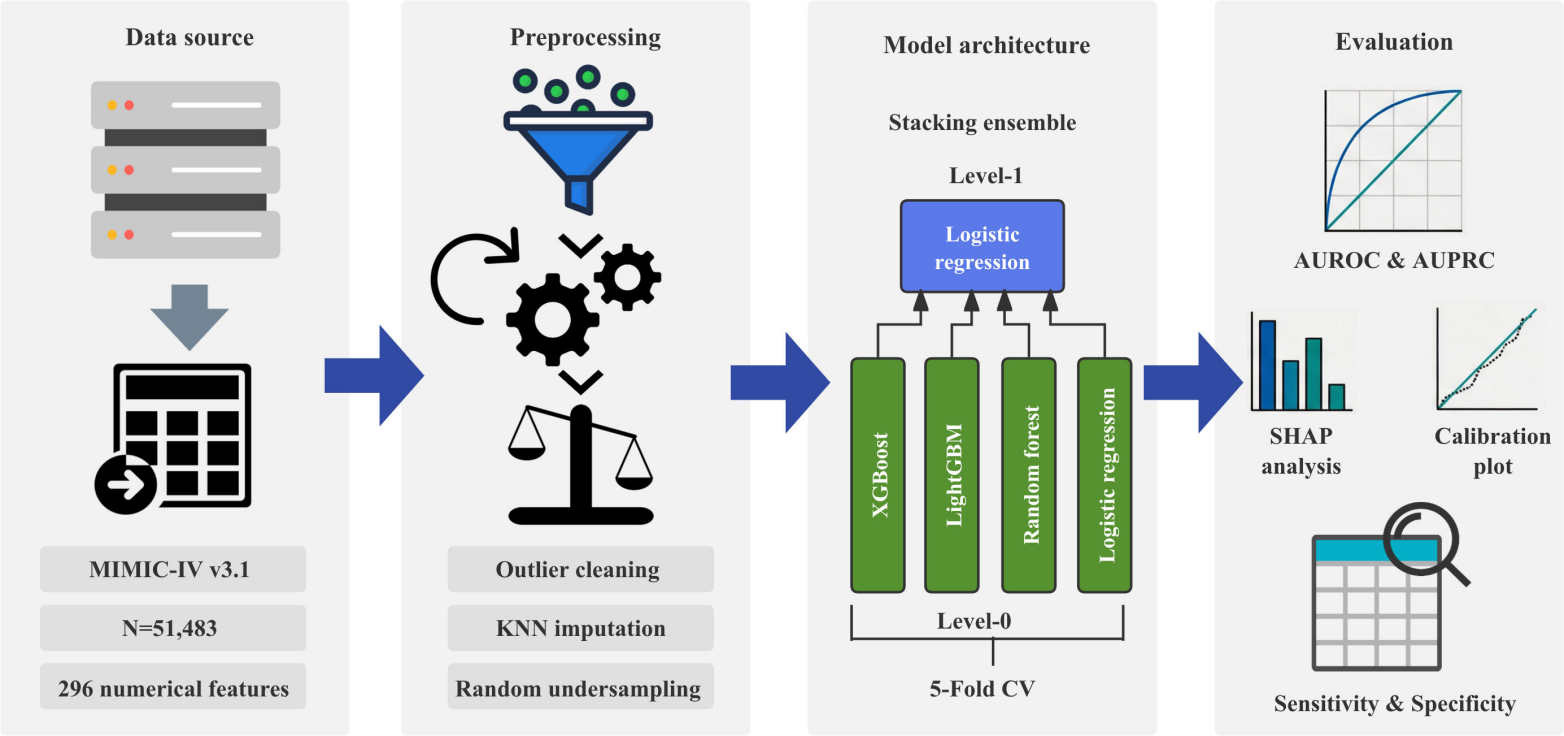
Overall experimental workflow and model development pipeline. Clinical data extracted from the MIMIC-IV v3.1 database yielded a final analytic cohort of 51,483 patients. After preprocessing, the final modeling matrix comprised 296 predictors, including one-hot encoded nominal categorical variables. The data preprocessing phase involved outlier cleaning, KNN imputation, and random undersampling to address class imbalance. Subsequently, a 2-level Stacking Ensemble model was constructed under a 5-fold cross-validation framework, using XGBoost, LightGBM, Random Forest, and Logistic Regression (LR) as base learners (level 0) to feed an LR metalearner (level 1). Finally, the predictive performance and clinical interpretability of the model were systematically evaluated using metrics including AUROC, AUPRC, and SHAP analysis and calibration plots. AUPRC: area under the precision-recall curve; AUROC: area under the receiver operating characteristic curve; CV: cross-validation; KNN: k-nearest neighbors; LightGBM: Light Gradient Boosting Machine; MIMIC-IV: Medical Information Mart for Intensive Care; SHAP: Shapley Additive Explanations; XGBoost: Extreme Gradient Boosting.

The final dataset was partitioned into a training set (80%) and an independent test set (20%) using stratified sampling. This approach ensured that the proportion of malnutrition cases remained consistent across splits. No data from the test set were used during the feature engineering, imputation fitting, or model training phases to strictly prevent data leakage. Figure 2 presents the methodological framework of this study.

### Model Development

The development of the predictive model was centered on a Stacking Ensemble Learning framework, termed E-NUTRIC, designed to integrate the strengths of diverse ML algorithms for robust risk stratification. This approach was selected to mitigate the limitations of single-model classifiers, which may suffer from overfitting or fail to capture complex nonlinear interactions within high-dimensional clinical data [23,24]. The ensemble architecture consisted of 2 layers: a base layer comprising 4 distinct algorithms and a metalearner layer that synthesized their outputs into a final calibrated probability.

The base layer comprised LR, RF, XGBoost, and LightGBM. LR was chosen as a linear baseline to capture straightforward additive relationships and provide interpretability [25]. RF, a bagging ensemble method, was used for its ability to reduce variance and handle high-dimensional feature spaces effectively [26,27]. XGBoost and LightGBM, both gradient boosting frameworks, were selected for their superior performance on tabular data and their capacity to model complex nonlinear dependencies through iterative error correction [28]. To ensure reproducibility and avoid the potential pitfalls of overfitting during exhaustive hyperparameter searching on a limited dataset, we used a set of fixed, predefined hyperparameters based on established practices for imbalanced clinical datasets rather than performing a grid search. For instance, tree-based models were configured with 500 estimators to ensure sufficient learning capacity, while regularization parameters were set to prevent overfitting. A detailed list of all hyperparameters for each algorithm is provided in Multimedia Appendix 3.

Class imbalance was handled exclusively using foldwise RUS within the training pipeline to prevent information leakage. To avoid mathematically distorting the base learners and causing severe overprediction, no additional algorithm-level class weighting was concurrently applied. Specifically, class weights were explicitly disabled for LR, RF, and the stacking metalearner; scale_pos_weight was dynamically set to 1 for XGBoost; and the is_unbalance flag was disabled for LightGBM.

The stacking process was implemented using a 5-fold cross-validation scheme within the training set. In this procedure, the training data were divided into 5 folds. For each fold, the base models were trained on the remaining 4 folds and then generated predictions for the holdout fold. These out-of-fold predictions from all 4 base learners were then concatenated to construct a new feature matrix, which served as the input for the metalearner. We selected LR as the metalearner to linearly combine the probabilistic outputs of the base models. This choice was motivated by the need for a calibrated and interpretable final score that effectively weighs the confidence of each base classifier. The metalearner was trained on the full set of out-of-fold predictions, enabling it to learn the optimal combination of base model outputs that minimized the overall prediction error.

The final E-NUTRIC model output was a continuous probability score ranging from 0 to 1, representing the risk of malnutrition. To facilitate clinical decision-making, a definitive classification was determined using a probability threshold of 0.5. No early stopping mechanisms were used during training to maintain consistency across all experiments. All modeling was implemented using Python (version 3.10; Python Software Foundation) with the scikit-learn, xgboost, and lightgbm libraries.

### Statistical Analysis and Model Evaluation

Baseline characteristics of the full analytic cohort were rigorously compared between patients with and with no malnutrition risk. Given the inherent long-tail, nonnormal distributions characteristic of large-scale, real-world EHR data, all continuous variables were uniformly summarized as medians with IQRs. Consequently, continuous physiological and laboratory parameters were compared using the nonparametric Mann-Whitney *U* test. Categorical variables—including binary clinical indicators, specific interventions, and one-hot encoded features—were expressed as absolute frequencies and percentages (n, %). Differences in categorical proportions were evaluated using the Pearson Chi-square test when the expected frequency in any cell of a 2×2 contingency table was less than 5. All statistical tests were 2-sided, and a *P* value of <.05 was considered statistically significant.

Model performance was comprehensively evaluated on the held-out test set using a suite of metrics designed for imbalanced classification tasks. The primary evaluation metric was the AUROC, which assesses the model’s discriminative ability across all possible thresholds. To rigorously compare the predictive performance of the E-NUTRIC model against the clinical baseline (mNUTRIC score) and individual ML algorithms, we used the DeLong test for correlated receiver operating characteristic curves [29]. In instances where the DeLong test was inconclusive or yielded unstable estimates due to sample size constraints, we used a nonparametric bootstrap method with 1000 resamples to estimate the CIs and statistical significance of the AUROC differences. Additionally, given the low prevalence of malnutrition in our cohort (4384/51,483, 8.5%), we prioritized the AUPRC as a key indicator of model performance, as it is less sensitive to class imbalance than AUROC [13]. We also reported standard classification metrics including Accuracy, Sensitivity, Specificity, and *F*_1_-score based on a probability threshold of 0.5.

To enhance the clinical utility and interpretability of the E-NUTRIC model, we integrated SHAP into our evaluation framework [30]. While stacking ensembles provide superior predictive performance, they lack native transparency because of their complex, heterogeneous architecture. To obtain interpretable and stable local explanations, SHAP analysis was applied specifically to the most predictive component of our ensemble—XGBoost, which was the best-performing individual tree-based base learner. This approach elucidates the critical nonlinear feature interactions driving the risk predictions without compromising computational efficiency. This analysis provided both global feature importance rankings and local explanations for individual patient predictions, offering transparency into the model’s decision-making process.

Furthermore, to assess the reliability of the model’s risk probabilities, we conducted a comprehensive calibration analysis. For the calibration analysis, mNUTRIC ordinal scores were first converted to predicted probabilities. This was achieved by fitting a univariate LR model on the training set and evaluating its outputs on the held-out test set. For our ML framework, because RUS intrinsically shifts the background prevalence, raw probability outputs from the final Stacking model inherently overestimate risk. Therefore, we performed a post hoc Platt recalibration. The recalibration model was fitted on an independent split within the training dataset and evaluated on the same test set, yielding the Recalibrated E-NUTRIC probabilities. The calibration curve serves as a critical diagnostic for identifying over- or underestimation biases in the predicted risks [31]. All statistical analyses and model development were performed using Python (version 3.10) with the scikit-learn, scipy, shap, and lifelines libraries.

### Ethical Considerations

The study was approved by the Institutional Review Board of the Beth Israel Deaconess Medical Center (2001-P-001699/14) and the Massachusetts Institute of Technology (no. 0403000206), and individual consent for this retrospective analysis was waived.

## Results

### Baseline Characteristics of the Study Population

The final study cohort comprised 51,483 patients, among whom 4384 (8.5%) were identified as being at high risk of malnutrition (Risk Group), while 47,099 (91.5%) were classified as being at no risk of malnutrition (No Risk Group). A summary of key demographic and clinical characteristics is presented in Table 1.

**Table 1.** Baseline demographic, anthropometric, clinical, vital sign, and laboratory characteristics of the full analytic cohort stratified by malnutrition risk status. A total of 51,483 patients were enrolled, of whom 47,099 were nonmalnourished and 4384 had malnutrition^a^.

Variable	Malnutrition risk group (n=4384)	No risk group (n=47,099)	*P* value
Demographics			
Age (years), median (IQR)	68.00 (57.00-78.00)	67.00 (56.00-78.00)	.002
Sex, n (%)			.02
Male	2436 (55.6)	27,030 (57.4)	
Female	1948 (44.4)	20,069 (42.6)	
Ethnicity, n (%)			<.001
Asian	147 (3.4)	1365 (2.9)	
Black	450 (10.3)	4073 (8.6)	
Hispanic	135 (3.1)	1653 (3.5)	
White	2801 (63.9)	30,974 (65.8)	
Other/Unknown	851 (19.4)	9034 (19.2)	
Anthropometrics			
BMI (kg/m²), median (IQR)	25.19 (20.99-30.06)	28.34 (24.76-32.89)	<.001
Weight (kg), median (IQR)	69.53 (57.30-85.00)	80.30 (68.00-95.35)	<.001
Clinical parameters and scores			
ICU^b^ type, n (%)			<.001
CCU^c^	324 (7.4)	5404 (11.5)	
CVICU^d^	208 (4.7)	10,418 (22.1)	
MICU^e^	1260 (28.7)	7990 (17.0)	
SICU^f^	2339 (53.4)	18,893 (40.1)	
Other^g^	253 (5.8)	4394 (9.3)	
APACHE II^h^ score, median (IQR)	22.00 (17.00-27.00)	18.00 (13.00-23.00)	<.001
SOFA^i^ score, median (IQR)	5.00 (3.00-8.00)	4.00 (2.00-6.00)	<.001
Charlson Comorbidity Index, median (IQR)	6.00 (4.00-8.00)	5.00 (3.00-7.00)	<.001
Pre-ICU length of stay (days), median (IQR)	0.09 (0.04-2.14)	0.08 (0.03-0.75)	<.001
Laboratory values (24 hours), mean			
Albumin (g/dL), median (IQR)	2.74 (2.37-3.14)	3.10 (2.70-3.50)	<.001
Creatinine (mg/dL), median (IQR)	0.91 (0.62-1.55)	0.90 (0.70-1.27)	.32
BUN^j^ (mg/dL), median (IQR)	23.55 (14.75-39.03)	18.25 (12.93-28.00)	<.001
Glucose (mg/dL) (Chemistry), median (IQR)	124.83 (108.51-152.05)	120.50 (106.75-142.76)	<.001
Potassium (mEq/L) (Chemistry), median (IQR)	4.06 (3.85-4.30)	4.08 (3.85-4.32)	.02
Lymphocytes (Absolute), median (IQR)	1.02 (0.64-1.52)	1.40 (0.89-2.04)	<.001
Phosphate (mg/dL), median (IQR)	3.37 (2.95-3.91)	3.30 (2.87-3.77)	<.001
Magnesium (mg/dL), median (IQR)	2.03 (1.92-2.17)	2.05 (1.93-2.20)	<.001
Cholesterol (mg/dL), median (IQR)	117.00 (86.00-152.75)	150.00 (119.00-185.00)	<.001

a Continuous variables are expressed as median (IQR) and compared using the Mann-Whitney *U* test. Categorical variables are expressed as counts and percentages, n (%), and compared using the chi-square test. *P* values are 2-tailed, and statistical significance was established at *P*<.05.

b ICU: intensive care unit.

c CCU: coronary care unit.

d CVICU: cardiovascular intensive care unit.

e MICU: medical intensive care unit.

f SICU: surgical intensive care unit.

g ICU type categories in Table 1 were partially aggregated for readability. The SICU category includes MICU/SICU, Neuro SICU, SICU, and TSICU; the Other category includes Neuro Intermediate, Neuro Stepdown, and Other. Detailed ICU subtype distributions are provided in Multimedia Appendix 4.

h APACHE II: Acute Physiology and Chronic Health Evaluation II.

i SOFA: Sequential Organ Failure Assessment.

j BUN: blood urea nitrogen.

The 2 groups were comparable in terms of age and gender distribution. However, significant disparities were observed in anthropometric measurements and clinical severity indices. Patients in the malnutrition risk group had a significantly lower BMI and lower admission weight. Clinical severity was substantially higher in the risk group, as indicated by elevated Acute Physiology and Chronic Health Evaluation II scores, Charlson Comorbidity Index, and SOFA scores. The mNUTRIC score was also significantly higher in the risk group.

Physiological parameters recorded within the first 24 hours further distinguished the 2 groups. The risk group demonstrated signs of hemodynamic instability, characterized by higher heart rates and respiratory rates. Laboratory analyses revealed significant nutritional and inflammatory differences; specifically, minimum serum albumin levels were lower in the risk group. Similarly, total cholesterol levels and lymphocyte counts were significantly reduced in the risk group (*P*<.001), while renal function markers such as blood urea nitrogen were elevated (*P*<.001). These findings indicate that the malnutrition risk group had a higher burden of critical illness and metabolic disturbances upon ICU admission. A comprehensive comparison of all analyzed features is provided in Multimedia Appendix 4.

### Predictive Performance Comparison

**Figure 3.**
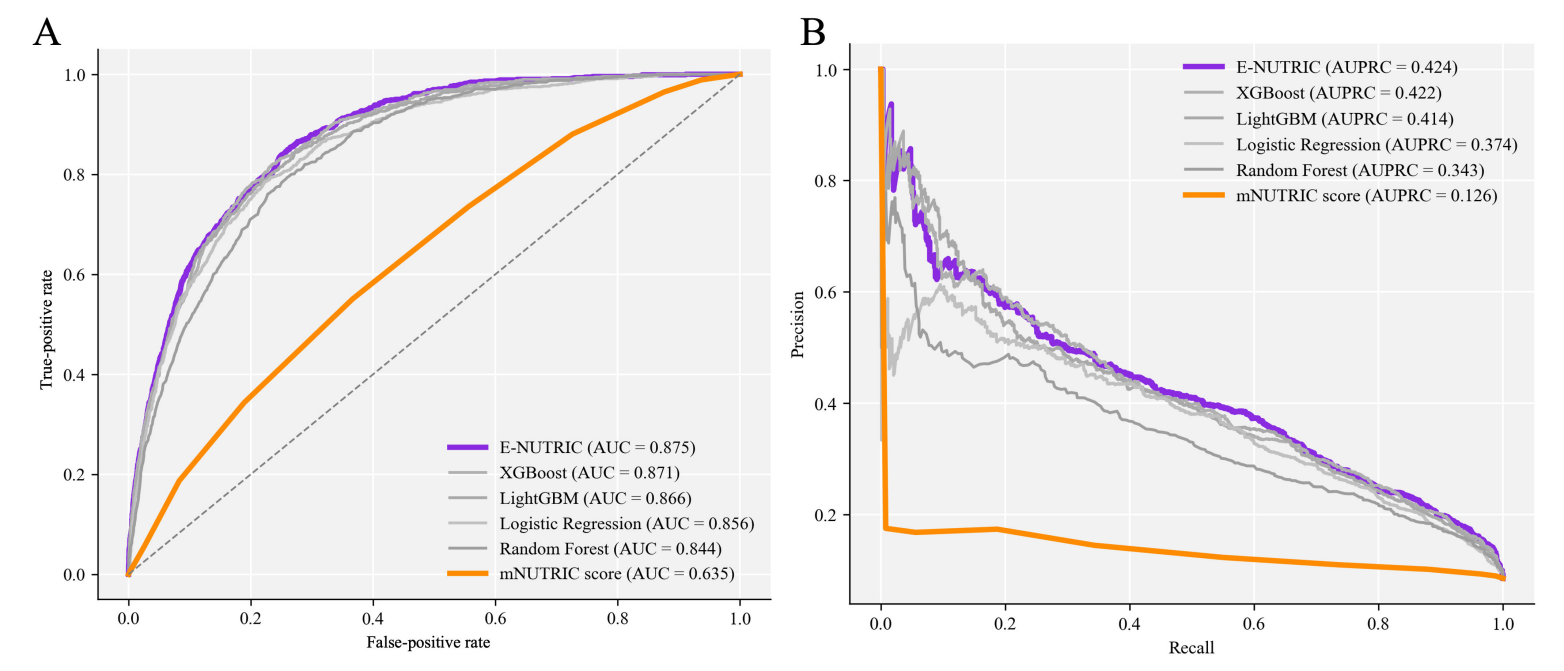
Discriminative performance of models and score. Receiver operating characteristic curves (A) and precision-recall curves (B). The proposed E-NUTRIC (purple line) significantly outperforms the clinical benchmark mNUTRIC score (orange line). Individual machine learning base learners (XGBoost, LightGBM, Random Forest, and Logistic Regression) are shown in light gray for comparison, demonstrating the superior performance of the ensemble approach. AUC: area under the receiver operating characteristic curve; AUPRC: area under the precision-recall curve; E-NUTRIC: Ensemble-NUTRIC; LightGBM: Light Gradient Boosting Machine; mNUTRIC: modified NUTRIC; XGBoost: Extreme Gradient Boosting.

The proposed E-NUTRIC model demonstrated superior predictive performance compared with both the existing clinical scoring system and the individual ML algorithms. As detailed in Table 2, the E-NUTRIC model achieved the highest discrimination with an AUROC of 0.875 (95% CI 0.864‐0.885). This represents a substantial and statistically significant improvement over the standard mNUTRIC score, which yielded an AUROC of 0.635 (95% CI 0.617‐0.652, *P*<.001). The receiver operating characteristic curves presented in Figure 3A illustrate this disparity, showing that the E-NUTRIC model maintains a higher true-positive rate across all decision thresholds compared with the linear clinical baseline.

In the context of the imbalanced dataset, the AUPRC provided a more informative assessment of model utility. The E-NUTRIC model achieved an AUPRC of 0.424, which is more than 3 times that of the mNUTRIC score. Figure 3 highlights this performance gap, showing that the ensemble model maintains precision better as recall increases, whereas the performance of the mNUTRIC score degrades rapidly, approaching the no-skill baseline. Among the individual base learners, XGBoost and LightGBM exhibited strong performance with AUROC of 0.871 and 0.866, respectively.

**Table 2.** Performance comparison of Ensemble-NUTRIC (E-NUTRIC) with baseline models and clinical scores on the test set.

Model	AUROC^a^ (95% CI)	AUPRC^b^	Accuracy	*F*_1_-score	Sensitivity	Specificity
E-NUTRIC	0.875 (0.864-0.885)^c^	0.424	0.785	0.384	0.790	0.784
LightGBM^d^	0.866 (0.854‐0.877)	0.414	0.770	0.372	0.800	0.767
XGBoost^e^	0.871 (0.860‐0.882)	0.422	0.774	0.380	0.812	0.770
LR^f^	0.856 (0.844‐0.869)	0.374	0.783	0.377	0.772	0.784
Random Forest	0.844 (0.831‐0.855)	0.343	0.723	0.334	0.813	0.715
mNUTRIC^g^ Score	0.635 (0.617‐0.652)	0.126	0.142	0.164	0.989	0.063

a AUROC: area under the receiver operating characteristic curve.

b AUPRC: area under the precision-recall curve.

c The proposed E-NUTRIC model demonstrated statistically significant improvement in AUROC compared with the clinical baseline mNUTRIC score (*P*<.001) and the best-performing single base learner, XGBoost (*P*=.018), evaluated via the DeLong test.

d LightGBM: Light Gradient Boosting Machine.

e XGBoost: Extreme Gradient Boosting.

f LR: Logistic Regression.

g mNUTRIC: modified NUTRIC.

At the standard probability threshold of 0.5, the E-NUTRIC model demonstrated a balanced classification profile with an accuracy of 0.785, a sensitivity of 0.790, and a specificity of 0.784. In contrast, the mNUTRIC score showed extremely high sensitivity but negligible specificity, indicating a tendency to overpredict risk and generate excessive false positives. The superior *F*_1_-score of the E-NUTRIC model versus mNUTRIC further underscores its robustness in correctly identifying true malnutrition cases while minimizing false positives. Detailed performance curves for all individual base models are provided in Multimedia Appendix 5.

### Model Interpretability and Feature Analysis

To elucidate the decision-making logic of the predictive framework, we used SHAP values to quantify the contribution of individual features. Given the heterogeneous architecture of the final stacking ensemble, SHAP was applied to its most predictive tree-based component—the XGBoost base learner—to ensure accurate mathematical attribution and computational stability.

**Figure 4.**
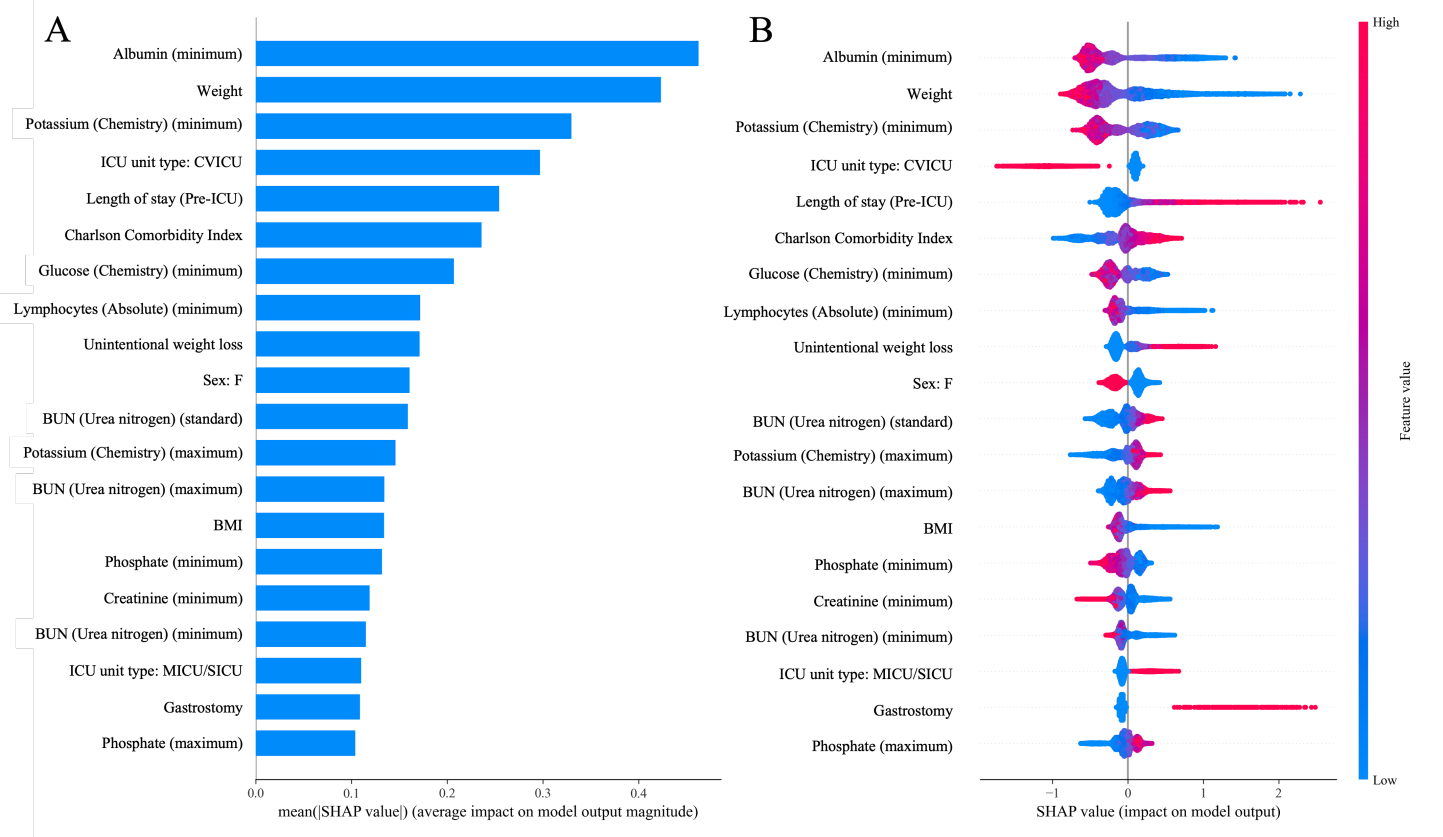
SHAP global feature importance and summary analysis derived from the XGBoost component. (A) The bar plot ranks the top 20 features by their mean absolute SHAP value, indicating their overall mathematical contribution to the model’s predictions. (B) The summary plot visualizes the distribution of SHAP values across individual patients (represented by dots). Note the color gradient: red indicates a high feature value, and blue indicates a low feature value. A positive SHAP value on the x-axis drives the model toward a higher predicted risk of malnutrition. BUN: blood urea nitrogen; CVICU: cardiovascular intensive care unit; ICU: intensive care unit; MICU: medical intensive care unit; SICU: surgical intensive care unit; SHAP: Shapley Additive Explanations.

Figures 4A and 4B present the global feature importance rankings alongside their directional impacts on the model’s output. Minimum serum albumin emerged as the most dominant predictor, followed strongly by admission weight, minimum potassium levels, and specific ICU admission location. The summary plot delineates distinct clinical phenotypes: lower physiological values (blue dots) of albumin, weight, and blood potassium are aggressively associated with positive SHAP values, increasing the predicted risk of malnutrition. Notably, admission to cardiovascular intensive care unit exerted a distinctly protective effect on the risk score, whereas prolonged pre-ICU length of stay and higher Charlson Comorbidity Index scores were strongly positively correlated with malnutrition risk. This adequately reflects the compounding impact of chronic disease burden and extended hospitalization on nutritional depletion.

**Figure 5.**
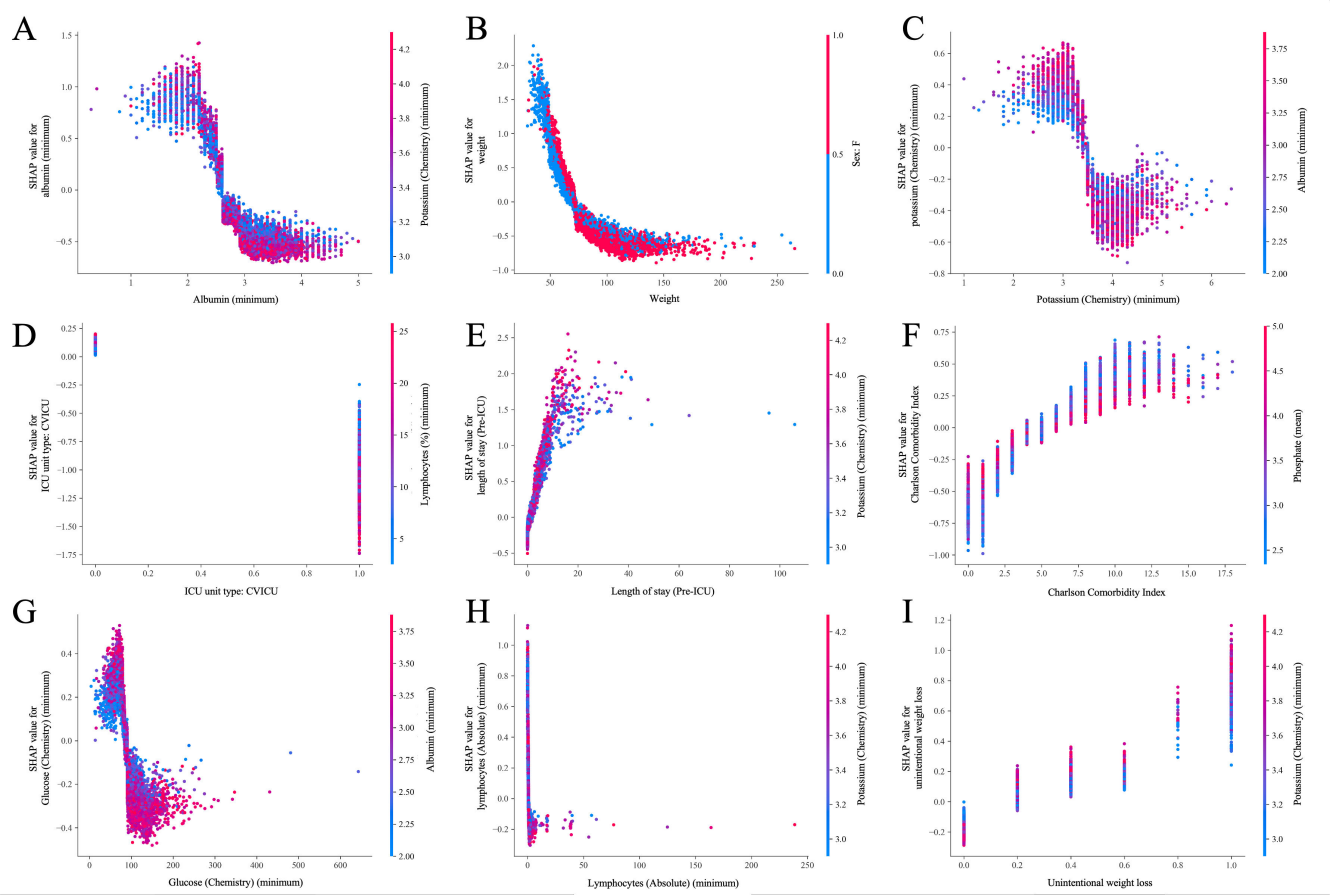
SHAP partial dependence plots showing nonlinear clinical thresholds for the top 9 features: (A) minimum albumin, (B) admission weight, (C) minimum potassium, (D) CVICU admission, (E) pre-ICU length of stay, (F) Charlson Comorbidity Index, (G) minimum glucose, (H) minimum absolute lymphocyte count, and (I) unintentional weight loss. These plots illustrate the marginal effect of each variable on predicted malnutrition risk. The x-axis represents the raw feature value from the electronic health record, and the y-axis represents the corresponding SHAP value. The color bar in each panel indicates the interacting feature value, illustrating multidimensional model behavior. Notable clinically relevant nonlinear thresholds are observed natively in albumin, weight, and absolute lymphocytes. CVICU: cardiovascular intensive care unit; ICU: intensive care unit; SHAP: Shapley Additive Explanations.

Figure 5A-I further delineate the granular, nonlinear relationships for the top 9 predictive features via SHAP dependence plots. A critical threshold is observed for serum albumin: the risk contribution escalates sharply as levels fall below approximately 2.8 g/dL, corroborating its clinical utility as a potent negative acute-phase reactant. Similarly, the weight dependence plot explicitly demonstrates a steep surge in risk for patients weighing less than 70 kg, which plateaus at higher baseline weights. Electrolyte and metabolic profiles reveal that acute physiological stress—specifically early hypokalemia (<3.5 mEq/L) and hypoglycemia (<100 mg/dL)—disproportionately compounds the predicted risk. Furthermore, severe early lymphopenia (low absolute lymphocytes) and the documented presence of unintentional weight loss exhibited definitive risk spikes. Collectively, these interpretability analyses confirm that the model effectively captured clinically and biologically plausible signals, accurately prioritizing established markers of severe catabolism, structural depletion, and diminished physiological reserve without relying on arbitrary data artifacts. Furthermore, a global SHAP interaction analysis (refer to Multimedia Appendix 6 for details) revealed strong second-order feature dependencies, most notably between absolute admission weight and female gender. This demonstrates the ensemble’s capacity to automatically learn sex-specific physiological thresholds for body mass, highlighting a critical advantage of nonlinear ML architectures over traditional additive clinical scores.

### Risk Stratification and Calibration Analysis

**Figure 6.**
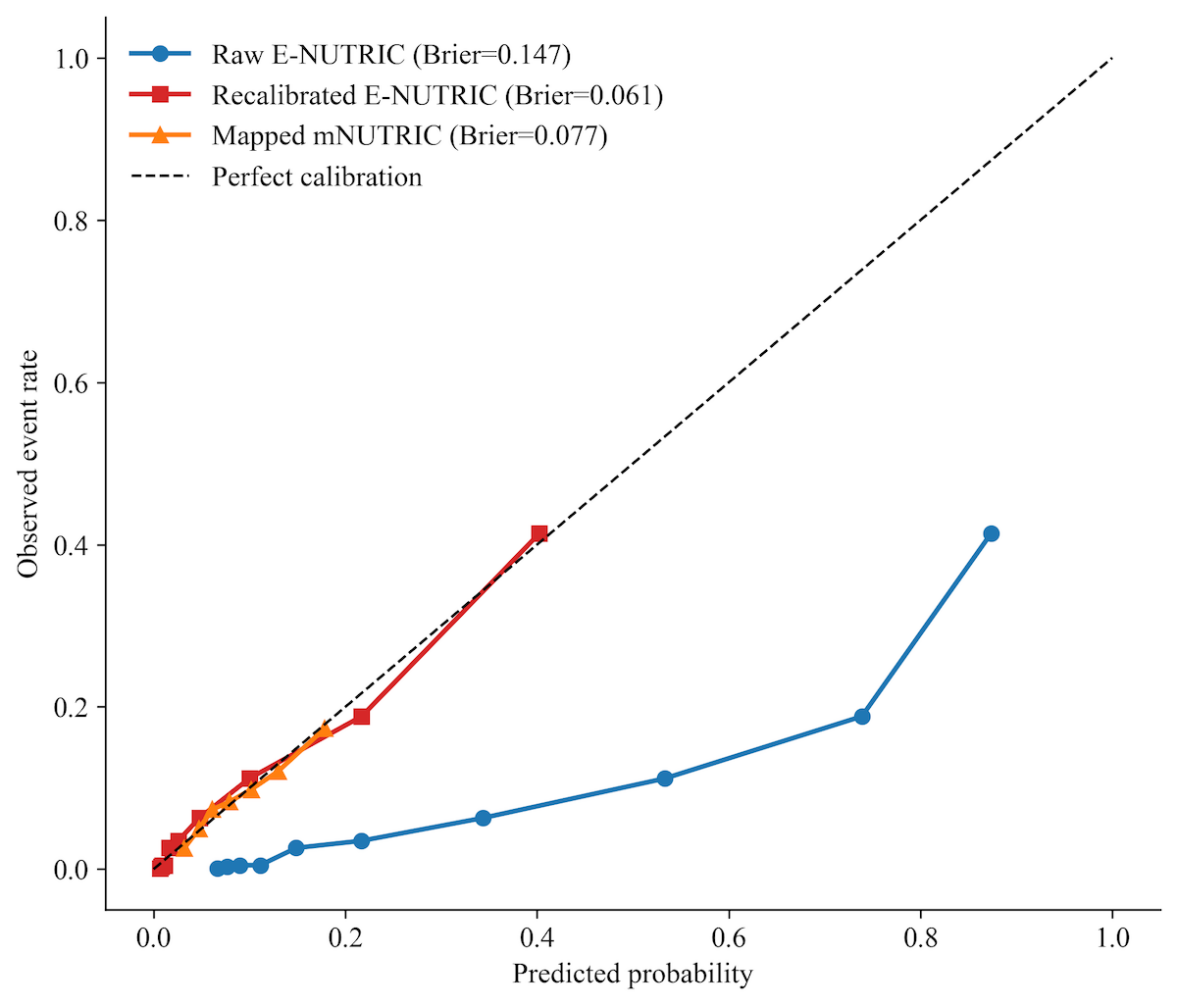
Calibration plots on the test set demonstrating dynamic risk stratification. The diagonal dashed gray line represents perfect calibration, where predicted probabilities perfectly match observed clinical frequencies. The Raw E-NUTRIC probabilities (blue circles) display the expected overestimation inherent to algorithms trained on artificially balanced resampled datasets. Following Platt scaling, the Recalibrated E-NUTRIC (red squares) rigorously corrects this shift, accurately mapping risk across the entire spectrum. The mapped mNUTRIC score (orange triangles)—derived via logistic transformation—is provided for clinical baseline comparison. Notably, while mNUTRIC predictions are severely compressed into the lower probability tier (<0.2), E-NUTRIC effectively spans the full dynamic range to identify critical, high-risk phenotypes. E-NUTRIC: Ensemble-NUTRIC; mNUTRIC: modified NUTRIC.

To evaluate the reliability of the risk predictions across the probability spectrum, a comprehensive calibration analysis was performed on the test set (Figure 6). As expected, the raw E-NUTRIC probabilities—generated directly from the ensemble trained on the RUS balanced dataset—exhibited structural overestimation of absolute risk, deviating systematically below the perfect calibration diagonal (Brier score=0.1471; expected calibration error [ECE]=0.2347). To correct this prevalence-induced probability shift, Platt scaling was applied. The resulting Recalibrated E-NUTRIC model demonstrated excellent alignment with true clinical event rates (Brier score=0.0615; ECE=0.0095), accurately tracking expected outcomes across all risk deciles.

Following recalibration, we compared the E-NUTRIC ensemble against the baseline mNUTRIC score. To ensure a mathematical equivalent comparison, the ordinal mNUTRIC scores were first mapped to continuous probabilities using a training-set logistic transformation (yielding a Brier score of 0.0765 and an ECE of 0.0045 on the test set). While the mapped mNUTRIC achieved tight calibration metrics, the resulting calibration curve highlights a fundamental limitation of the traditional clinical scoring system: its predicted probabilities are severely compressed, clustering almost entirely below a threshold of 0.20. This indicates that the conventional score lacks the dynamic range necessary to differentiate between moderate-risk and genuinely high-risk patients, effectively categorizing the entire cohort into a low-probability band. In stark contrast, the Recalibrated E-NUTRIC model uses the full probability space. Unlike the mNUTRIC score, which plateaus prematurely, our model effectively isolates a high-risk subgroup of patients at the upper tail of the distribution, providing clinicians with a highly discriminative and granular tool for identifying patients who acutely require intensive nutritional interventions.

## Discussion

### Principal Findings

In this study, we developed and internally validated the E-NUTRIC model, a stacking ensemble ML framework designed to predict malnutrition risk among critically ill patients using routine EHR data. Our evaluation indicates that the E-NUTRIC ensemble provides superior discriminative capacity compared with the traditional mNUTRIC score, achieving an AUROC of 0.875 compared with 0.635 for the clinical baseline. Notably, in the context of the naturally imbalanced prevalence of ICU malnutrition, the model yielded a substantial, more than 3-fold increase in the AUPRC. This enhancement addresses a recurring limitation in existing additive clinical scoring systems, which frequently lack the dynamic range necessary to effectively stratify patients at the upper end of the risk spectrum.

While individual tree-based base learners such as XGBoost and LightGBM also exhibited robust performance, the level 1 stacking strategy provided incremental improvement in the stabilization of predictive reliability. This supports the premise that integrating diverse functional algorithms can effectively synthesize complementary clinical signals within complex datasets. Furthermore, to ensure accurate mathematical attribution and avoid the methodological artifacts associated with explaining metalearners, we applied SHAP analysis specifically to the highly predictive XGBoost component. This analytical approach elucidated the nonlinear feature relationships driving the predictions, identifying minimum serum albumin, admission weight, early hypokalemia, and specific ICU unit types as the most influential predictors of malnutrition risk.

### Comparison With Prior Work

Previous research in nutritional assessment has predominantly relied on static scoring systems such as the Geriatric Nutrition Risk Index [32], the Controlling Nutrition Status score [33], and the Subjective Global Assessment [9]. While effective in general wards or outpatient oncology settings [34,35], these tools often fail to account for the rapid physiological changes characteristic of the ICU environment. The mNUTRIC score was specifically designed for critically ill patients, yet our findings align with recent studies suggesting its limited discriminatory power without the inclusion of inflammatory markers such as interleukin-6, which are rarely routinely available [36].

In the field of clinical artificial intelligence, recent efforts have applied deep learning and time series analysis to predict patient deterioration [37,38]. However, many of these models prioritize raw predictive accuracy over interpretability and often involve substantial computational demands that limit bedside deployment. Our study differs from such approaches by using a stacking ensemble approach on aggregated clinical features. Unlike single-model approaches or complex deep neural networks, our framework strikes a balance between predictive power and computational efficiency. The superior performance of E-NUTRIC over the mNUTRIC score validates the assertion that nonlinear modeling techniques are necessary to capture the complex interactions between physiological stress, organ failure, and nutritional depletion [39].

### Clinical Implications and Biological Plausibility

The interpretability analysis via SHAP values not only confirms the model’s reliance on clinically relevant features but also provides biological validation of the algorithm’s learning process. Minimum serum albumin emerged as the single most important predictor. Although traditionally viewed as a nutritional marker, hypoalbuminemia in the ICU is a potent indicator of the inflammatory response and capillary leak syndrome, both of which accelerate catabolism and nutritional depletion [40]. The sharp increase in risk when albumin falls below 2.8 g/dL, as observed in our partial dependence plots, aligns with the threshold often cited for severe malnutrition-inflammation complex syndrome.

Similarly, the model identified admission weight and minimum potassium levels as critical features. The nonlinear relationship observed with absolute admission weight—where risk escalates rapidly below 70 kg—was shown in our interaction analysis to be strongly modulated by female gender, dynamically capturing the phenotype of the frail patient with sarcopenia who lacks the metabolic reserve to withstand critical illness [41]. Beyond electrolytes such as potassium—which capture signals related to severe fluid-electrolyte shifts [42]—the model effectively leveraged granular one-hot encoded features, identifying the distinct protective baseline status of cardiovascular intensive care unit admissions and the compounding risk of documented unintentional weight loss and early severe lymphopenia. Unlike the mNUTRIC score, which is heavily weighted by the SOFA score and thus primarily reflects organ failure, E-NUTRIC incorporates these metabolic and anthropometric granularities, offering a more holistic view of the patient’s nutritional status.

Furthermore, the calibration analysis revealed a crucial clinical utility finding: the mNUTRIC score effectively “caps” risk prediction at approximately 20%, treating the vast majority of patients as low risk. In stark contrast, following Platt scaling correction for prevalence-induced shifts, the recalibrated E-NUTRIC model accurately spans the full dynamic probability spectrum. This capability allows for the identification of a high-risk cohort that would otherwise be missed by standard screening, enabling clinicians to direct aggressive nutritional interventions—such as early parenteral nutrition or high-protein delivery—to the patients who are most likely to benefit, thereby optimizing resource allocation.

### Limitations

Despite these promising results, our study is subject to several limitations. First, the model was developed and validated using the MIMIC-IV database, which represents a single-center cohort at the US tertiary care center. While the sample size is large and diverse, the care protocols and patient demographics may not fully generalize to community hospitals or different health care systems globally. To mitigate this, we used a robust cross-validation scheme and a holdout test set, but external validation on datasets such as the eICU Collaborative Research Database [43] remains a necessary next step to confirm generalizability.

Second, the definition of malnutrition in our study relied on *ICD-10* diagnostic codes. While this is a standard approach in large-scale EHR research, administrative codes typically have high specificity but low sensitivity, potentially leading to an underestimation of malnutrition prevalence. We attempted to address the resulting class imbalance by using RUS and optimizing for AUPRC, yet some in the control group patients may have had undiagnosed malnutrition. Future studies should ideally use prospectively collected data based on the Global Leadership Initiative on Malnutrition criteria to provide a more rigorous ground truth [44].

Third, the retrospective nature of the study necessitates the use of imputation for missing data. Although our preliminary experiments demonstrated that KNN imputation preserved data integrity better than other methods, any imputation introduces a degree of synthetic variability. We mitigated this by selecting KNN imputation based on preliminary experiments, but the potential for bias cannot be entirely eliminated.

Fourth, while SHAP values provide powerful associations, they do not imply causality. The feature importance rankings indicate which variables drive the prediction, but they do not prove that modifying a feature (eg, correcting hypokalemia) will reduce malnutrition risk. Clinical judgment remains essential in interpreting these outputs [45].

### Future Directions

Building on the E-NUTRIC model, future research should focus on prospective validation in a multicenter clinical setting. Integrating this model into an EHR-based clinical decision support system would allow evaluation of its real-world impact on clinical workflow and patient outcomes. Specifically, a randomized controlled trial comparing model-guided nutritional intervention versus standard care would be the gold standard for establishing clinical utility. Additionally, future iterations of the model could incorporate unstructured data, such as nursing notes or dietary intake logs, using natural language processing to further enhance predictive accuracy [46].

### Conclusions

This study presents E-NUTRIC, a stacking ensemble ML model designed for the early prediction of malnutrition risk in ICU patients. By integrating robust predictive algorithms with SHAP-based interpretability, E-NUTRIC offers an interpretable approach to risk stratification. Specifically, compared with the mNUTRIC score, the proposed model demonstrates superior discriminative performance and provides a finer-grained probability spectrum, effectively capturing key metabolic, anthropometric, and demographic features associated with malnutrition. Although prospective external validation remains necessary, E-NUTRIC demonstrates substantial potential as a clinical decision support tool to facilitate timely, targeted, and accurate nutritional interventions for critically ill patients at high risk.

## Supplementary material

10.2196/77872 Multimedia Appendix 1 Comprehensive data dictionary of the 296 predictors included in the Ensemble-NUTRIC analytic matrix.

10.2196/77872 Multimedia Appendix 2 Optimization of data preprocessing strategies.

10.2196/77872 Multimedia Appendix 3 Hyperparameter settings and model configuration.

10.2196/77872 Multimedia Appendix 4 Comprehensive baseline characteristics and feature statistics.

10.2196/77872 Multimedia Appendix 5 Comprehensive model performance analysis.

10.2196/77872 Multimedia Appendix 6 Shapley Additive Explanations global feature interaction network plot.

## Abbreviations

AUPRC: area under the precision-recall curve
AUROC: area under the receiver operating characteristic curve
E-NUTRIC: Ensemble-NUTRIC
ECE: expected calibration error
EHR: electronic health record
*ICD-10*: *International Statistical Classification of Diseases, Tenth Revision*
*ICD-9*: *International Classification of Diseases, Ninth Revision*
ICU: intensive care unit
KNN: k-nearest neighbor
LightGBM: Light Gradient Boosting Machine
LR: Logistic Regression
MIMIC-IV: Medical Information Mart for Intensive Care
ML: machine learning
mNUTRIC: modified NUTRIC
RF: Random Forest
RUS: random undersampling
SHAP: Shapley Additive Explanations
SOFA: Sequential Organ Failure Assessment
XGBoost: Extreme Gradient Boosting
