# Supplementary material for: Development and Interpretability Analysis of a Stacking Ensemble Model for Early Prediction of Nutritional Risk in Intensive Care Unit Patients: Retrospective Cohort Study
Source: JMIR Med Inform. 2026 Jun 3;14:e77872. doi: 10.2196/77872 (PMC13232782; doi:10.2196/77872)
Supplement: Multimedia Appendix 1 [file medinform-v14-e77872-s001.docx]

Multimedia Appendix 1: Comprehensive Data Dictionary of the 296 Predictors Included in the E-NUTRIC Analytic Matrix

Table S1. Comprehensive Feature List

This appendix enumerates the exact 296 features utilized by the machine learning algorithms. To prevent the arbitrary mathematical scaling of nominal categorical variables (such as ICU unit type, race, language, and marital status), these variables were transformed into independent binary continuous features using strict one-hot encoding. Notably, to preserve potential predictive patterns associated with the absence of clinical documentation, missing values in these nominal categories were explicitly encoded as separate indicator columns using the dummy_na=True framework. Additionally, to accurately reflect the true engineered feature matrix, certain vital signs may appear twice. These represent mathematically distinct variables extracted from different raw EHR tables: one set captured as static admission summaries (explicitly labeled as “Static EMR Summary”) and another calculated from high-granularity time-series events (e.g., “mean”). Pre-ICU Length of Stay (days) was defined as the interval between hospital admission and ICU admission. Following this preprocessing and feature expansion strategy, the final analytic matrix utilized for model training and evaluation comprised 296 distinct features.

| feature_name | | feature_type | | encoding_method | | included_in_final_model |
| --- | --- | --- | --- | --- | --- | --- |
| age | | numeric | | none | | Yes |
| weight | | numeric | | none | | Yes |
| height | | numeric | | none | | Yes |
| vitalhr (Static EMR Summary) | | numeric | | none | | Yes |
| vitalnbps (Static EMR Summary) | | numeric | | none | | Yes |
| vitalnbpd (Static EMR Summary) | | numeric | | none | | Yes |
| vitalnbpm (Static EMR Summary) | | numeric | | none | | Yes |
| vitalabps (Static EMR Summary) | | numeric | | none | | Yes |
| vitalabpd (Static EMR Summary) | | numeric | | none | | Yes |
| vitalabpm (Static EMR Summary) | | numeric | | none | | Yes |
| vitalrr (Static EMR Summary) | | numeric | | none | | Yes |
| vitalspo2 (Static EMR Summary) | | numeric | | none | | Yes |
| vitaltemperature (Static EMR Summary) | | numeric | | none | | Yes |
| lab_24hour_dailyweight | | numeric | | none | | Yes |
| lab_24hour_unintentionalweightloss | | numeric | | none | | Yes |
| inputnorepinephrine | | numeric | | none | | Yes |
| inputnorepinephrine_amount | | numeric | | none | | Yes |
| inputepinephrine | | numeric | | none | | Yes |
| inputepinephrine_amount | | numeric | | none | | Yes |
| inputvasopressin | | numeric | | none | | Yes |
| inputvasopressin_amount | | numeric | | none | | Yes |
| enteralnutrition | | numeric | | none | | Yes |
| enteralnutrition_amount | | numeric | | none | | Yes |
| parenteralnutrition | | numeric | | none | | Yes |
| parenteralnutrition_amount | | numeric | | none | | Yes |
| specialcomponents | | numeric | | none | | Yes |
| specialcomponents_amount | | numeric | | none | | Yes |
| rxglucocorticoids | | numeric | | none | | Yes |
| rxglucocorticoidstotalval | | numeric | | none | | Yes |
| metoclopramide | | numeric | | none | | Yes |
| metoclopramidetotalval | | numeric | | none | | Yes |
| supplements | | numeric | | none | | Yes |
| supplementstotalval | | numeric | | none | | Yes |
| insulin | | numeric | | none | | Yes |
| insulintotalval | | numeric | | none | | Yes |
| hormone | | numeric | | none | | Yes |
| hormonetotalval | | numeric | | none | | Yes |
| gastrostomy | | numeric | | none | | Yes |
| ventilation | | numeric | | none | | Yes |
| dialysis | | numeric | | none | | Yes |
| bypass | | numeric | | none | | Yes |
| aps_score | | numeric | | none | | Yes |
| age_score | | numeric | | none | | Yes |
| chronic_score | | numeric | | none | | Yes |
| apache_score | | numeric | | none | | Yes |
| sofa | | numeric | | none | | Yes |
| apsiii | | numeric | | none | | Yes |
| sirs | | numeric | | none | | Yes |
| sapsii | | numeric | | none | | Yes |
| oasis | | numeric | | none | | Yes |
| gcs | | numeric | | none | | Yes |
| charlson | | numeric | | none | | Yes |
| pre-ICU Length of Stay(days) | | numeric | | none | | Yes |
| bmi | | numeric | | none | | Yes |
| Diastolic BP (mean) | | numeric | | none | | Yes |
| Heart Rate (mean) | | numeric | | none | | Yes |
| mean BP (mean) | | numeric | | none | | Yes |
| Resp Rate (mean) | | numeric | | none | | Yes |
| SpO2 (mean) | | numeric | | none | | Yes |
| Systolic BP (mean) | | numeric | | none | | Yes |
| Temperature (mean) | | numeric | | none | | Yes |
| Diastolic BP (std) | | numeric | | none | | Yes |
| Heart Rate (std) | | numeric | | none | | Yes |
| mean BP (std) | | numeric | | none | | Yes |
| Resp Rate (std) | | numeric | | none | | Yes |
| SpO2 (std) | | numeric | | none | | Yes |
| Systolic BP (std) | | numeric | | none | | Yes |
| Temperature (std) | | numeric | | none | | Yes |
| Diastolic BP (first) | | numeric | | none | | Yes |
| Heart Rate (first) | | numeric | | none | | Yes |
| mean BP (first) | | numeric | | none | | Yes |
| Resp Rate (first) | | numeric | | none | | Yes |
| SpO2 (first) | | numeric | | none | | Yes |
| Systolic BP (first) | | numeric | | none | | Yes |
| Temperature (first) | | numeric | | none | | Yes |
| Diastolic BP (last) | | numeric | | none | | Yes |
| Heart Rate (last) | | numeric | | none | | Yes |
| mean BP (last) | | numeric | | none | | Yes |
| Resp Rate (last) | | numeric | | none | | Yes |
| SpO2 (last) | | numeric | | none | | Yes |
| Systolic BP (last) | | numeric | | none | | Yes |
| Temperature (last) | | numeric | | none | | Yes |
| Diastolic BP (min) | | numeric | | none | | Yes |
| Heart Rate (min) | | numeric | | none | | Yes |
| mean BP (min) | | numeric | | none | | Yes |
| Resp Rate (min) | | numeric | | none | | Yes |
| SpO2 (min) | | numeric | | none | | Yes |
| Systolic BP (min) | | numeric | | none | | Yes |
| Temperature (min) | | numeric | | none | | Yes |
| Diastolic BP (max) | | numeric | | none | | Yes |
| Heart Rate (max) | | numeric | | none | | Yes |
| mean BP (max) | | numeric | | none | | Yes |
| Resp Rate (max) | | numeric | | none | | Yes |
| SpO2 (max) | | numeric | | none | | Yes |
| Systolic BP (max) | | numeric | | none | | Yes |
| Temperature (max) | | numeric | | none | | Yes |
| Glucose (Blood Gas) (mean) | | numeric | | none | | Yes |
| Lactate (mean) | | numeric | | none | | Yes |
| Potassium (Blood Gas) (mean) | | numeric | | none | | Yes |
| Albumin (mean) | | numeric | | none | | Yes |
| Protein (Chemistry) (mean) | | numeric | | none | | Yes |
| Cholesterol (mean) | | numeric | | none | | Yes |
| Creatinine (mean) | | numeric | | none | | Yes |
| Glucose (Chemistry) (mean) | | numeric | | none | | Yes |
| Magnesium (mean) | | numeric | | none | | Yes |
| Phosphate (mean) | | numeric | | none | | Yes |
| Potassium (Chemistry) (mean) | | numeric | | none | | Yes |
| Total Protein (Chemistry) (mean) | | numeric | | none | | Yes |
| Transferrin (mean) | | numeric | | none | | Yes |
| Triglycerides (mean) | | numeric | | none | | Yes |
| BUN (Urea Nitrogen) (mean) | | numeric | | none | | Yes |
| Lymphocytes (Absolute) (mean) | | numeric | | none | | Yes |
| Lymphocytes (%) (mean) | | numeric | | none | | Yes |
| Erythrocyte Sedimentation Rate (mean) | | numeric | | none | | Yes |
| Glucose (Blood Gas) (std) | | numeric | | none | | Yes |
| Lactate (std) | | numeric | | none | | Yes |
| Potassium (Blood Gas) (std) | | numeric | | none | | Yes |
| Albumin (std) | | numeric | | none | | Yes |
| Protein (Chemistry) (std) | | numeric | | none | | Yes |
| Cholesterol (std) | | numeric | | none | | Yes |
| Creatinine (std) | | numeric | | none | | Yes |
| Glucose (Chemistry) (std) | | numeric | | none | | Yes |
| Magnesium (std) | | numeric | | none | | Yes |
| Phosphate (std) | | numeric | | none | | Yes |
| Potassium (Chemistry) (std) | | numeric | | none | | Yes |
| Total Protein (Chemistry) (std) | | numeric | | none | | Yes |
| Transferrin (std) | | numeric | | none | | Yes |
| Triglycerides (std) | | numeric | | none | | Yes |
| BUN (Urea Nitrogen) (std) | | numeric | | none | | Yes |
| Lymphocytes (Absolute) (std) | | numeric | | none | | Yes |
| Lymphocytes (%) (std) | | numeric | | none | | Yes |
| Erythrocyte Sedimentation Rate (std) | | numeric | | none | | Yes |
| Glucose (Blood Gas) (first) | | numeric | | none | | Yes |
| Lactate (first) | | numeric | | none | | Yes |
| Potassium (Blood Gas) (first) | | numeric | | none | | Yes |
| Albumin (first) | | numeric | | none | | Yes |
| Protein (Chemistry) (first) | | numeric | | none | | Yes |
| Cholesterol (first) | | numeric | | none | | Yes |
| Creatinine (first) | | numeric | | none | | Yes |
| Glucose (Chemistry) (first) | | numeric | | none | | Yes |
| Magnesium (first) | | numeric | | none | | Yes |
| Phosphate (first) | | numeric | | none | | Yes |
| Potassium (Chemistry) (first) | | numeric | | none | | Yes |
| Total Protein (Chemistry) (first) | | numeric | | none | | Yes |
| Transferrin (first) | | numeric | | none | | Yes |
| Triglycerides (first) | | numeric | | none | | Yes |
| BUN (Urea Nitrogen) (first) | | numeric | | none | | Yes |
| Lymphocytes (Absolute) (first) | | numeric | | none | | Yes |
| Lymphocytes (%) (first) | | numeric | | none | | Yes |
| Erythrocyte Sedimentation Rate (first) | | numeric | | none | | Yes |
| Glucose (Blood Gas) (last) | | numeric | | none | | Yes |
| Lactate (last) | | numeric | | none | | Yes |
| Potassium (Blood Gas) (last) | | numeric | | none | | Yes |
| Albumin (last) | | numeric | | none | | Yes |
| Protein (Chemistry) (last) | | numeric | | none | | Yes |
| Cholesterol (last) | | numeric | | none | | Yes |
| Creatinine (last) | | numeric | | none | | Yes |
| Glucose (Chemistry) (last) | | numeric | | none | | Yes |
| Magnesium (last) | | numeric | | none | | Yes |
| Phosphate (last) | | numeric | | none | | Yes |
| Potassium (Chemistry) (last) | | numeric | | none | | Yes |
| Total Protein (Chemistry) (last) | | numeric | | none | | Yes |
| Transferrin (last) | | numeric | | none | | Yes |
| Triglycerides (last) | | numeric | | none | | Yes |
| BUN (Urea Nitrogen) (last) | | numeric | | none | | Yes |
| Lymphocytes (Absolute) (last) | | numeric | | none | | Yes |
| Lymphocytes (%) (last) | | numeric | | none | | Yes |
| Erythrocyte Sedimentation Rate (last) | | numeric | | none | | Yes |
| Glucose (Blood Gas) (min) | | numeric | | none | | Yes |
| Lactate (min) | | numeric | | none | | Yes |
| Potassium (Blood Gas) (min) | | numeric | | none | | Yes |
| Albumin (min) | | numeric | | none | | Yes |
| Protein (Chemistry) (min) | | numeric | | none | | Yes |
| Cholesterol (min) | | numeric | | none | | Yes |
| Creatinine (min) | | numeric | | none | | Yes |
| Glucose (Chemistry) (min) | | numeric | | none | | Yes |
| Magnesium (min) | | numeric | | none | | Yes |
| Phosphate (min) | | numeric | | none | | Yes |
| Potassium (Chemistry) (min) | | numeric | | none | | Yes |
| Total Protein (Chemistry) (min) | | numeric | | none | | Yes |
| Transferrin (min) | | numeric | | none | | Yes |
| Triglycerides (min) | | numeric | | none | | Yes |
| BUN (Urea Nitrogen) (min) | | numeric | | none | | Yes |
| Lymphocytes (Absolute) (min) | | numeric | | none | | Yes |
| Lymphocytes (%) (min) | | numeric | | none | | Yes |
| Erythrocyte Sedimentation Rate (min) | | numeric | | none | | Yes |
| Glucose (Blood Gas) (max) | | numeric | | none | | Yes |
| Lactate (max) | | numeric | | none | | Yes |
| Potassium (Blood Gas) (max) | | numeric | | none | | Yes |
| Albumin (max) | | numeric | | none | | Yes |
| Protein (Chemistry) (max) | | numeric | | none | | Yes |
| Cholesterol (max) | | numeric | | none | | Yes |
| Creatinine (max) | | numeric | | none | | Yes |
| Glucose (Chemistry) (max) | | numeric | | none | | Yes |
| Magnesium (max) | | numeric | | none | | Yes |
| Phosphate (max) | | numeric | | none | | Yes |
| Potassium (Chemistry) (max) | | numeric | | none | | Yes |
| Total Protein (Chemistry) (max) | | numeric | | none | | Yes |
| Transferrin (max) | | numeric | | none | | Yes |
| Triglycerides (max) | | numeric | | none | | Yes |
| BUN (Urea Nitrogen) (max) | | numeric | | none | | Yes |
| Lymphocytes (Absolute) (max) | | numeric | | none | | Yes |
| Lymphocytes (%) (max) | | numeric | | none | | Yes |
| Erythrocyte Sedimentation Rate (max) | | numeric | | none | | Yes |
| gender_F | | binary_one_hot | | one_hot_dummy_na_true | | Yes |
| gender_M | | binary_one_hot | | one_hot_dummy_na_true | | Yes |
| gender_nan | | binary_one_hot | | one_hot_dummy_na_true | | Yes |
| race_AMERICAN INDIAN/ALASKA NATIVE | | binary_one_hot | | one_hot_dummy_na_true | | Yes |
| race_ASIAN | | binary_one_hot | | one_hot_dummy_na_true | | Yes |
| race_ASIAN - ASIAN INDIAN | | binary_one_hot | | one_hot_dummy_na_true | | Yes |
| race_ASIAN - CHINESE | | binary_one_hot | | one_hot_dummy_na_true | | Yes |
| race_ASIAN - KOREAN | | binary_one_hot | | one_hot_dummy_na_true | | Yes |
| race_ASIAN - SOUTH EAST ASIAN | | binary_one_hot | | one_hot_dummy_na_true | | Yes |
| race_BLACK/AFRICAN | | binary_one_hot | | one_hot_dummy_na_true | | Yes |
| race_BLACK/AFRICAN AMERICAN | | binary_one_hot | | one_hot_dummy_na_true | | Yes |
| race_BLACK/CAPE VERDEAN | | binary_one_hot | | one_hot_dummy_na_true | | Yes |
| race_BLACK/CARIBBEAN ISLAND | | binary_one_hot | | one_hot_dummy_na_true | | Yes |
| race_HISPANIC OR LATINO | | binary_one_hot | | one_hot_dummy_na_true | | Yes |
| race_HISPANIC/LATINO - CENTRAL AMERICAN | | binary_one_hot | | one_hot_dummy_na_true | | Yes |
| race_HISPANIC/LATINO - COLUMBIAN | | binary_one_hot | | one_hot_dummy_na_true | | Yes |
| race_HISPANIC/LATINO - CUBAN | | binary_one_hot | | one_hot_dummy_na_true | | Yes |
| race_HISPANIC/LATINO - DOMINICAN | | binary_one_hot | | one_hot_dummy_na_true | | Yes |
| race_HISPANIC/LATINO - GUATEMALAN | | binary_one_hot | | one_hot_dummy_na_true | | Yes |
| race_HISPANIC/LATINO - HONDURAN | | binary_one_hot | | one_hot_dummy_na_true | | Yes |
| race_HISPANIC/LATINO - MEXICAN | | binary_one_hot | | one_hot_dummy_na_true | | Yes |
| race_HISPANIC/LATINO - PUERTO RICAN | | binary_one_hot | | one_hot_dummy_na_true | | Yes |
| race_HISPANIC/LATINO - SALVADORAN | | binary_one_hot | | one_hot_dummy_na_true | | Yes |
| race_MULTIPLE RACE/ETHNICITY | | binary_one_hot | | one_hot_dummy_na_true | | Yes |
| race_NATIVE HAWAIIAN OR OTHER PACIFIC ISLANDER | | binary_one_hot | | one_hot_dummy_na_true | | Yes |
| race_OTHER | | binary_one_hot | | one_hot_dummy_na_true | | Yes |
| race_PATIENT DECLINED TO ANSWER | | binary_one_hot | | one_hot_dummy_na_true | | Yes |
| race_PORTUGUESE | | binary_one_hot | | one_hot_dummy_na_true | | Yes |
| race_SOUTH AMERICAN | | binary_one_hot | | one_hot_dummy_na_true | | Yes |
| race_UNABLE TO OBTAIN | | binary_one_hot | | one_hot_dummy_na_true | | Yes |
| race_UNKNOWN | | binary_one_hot | | one_hot_dummy_na_true | | Yes |
| race_WHITE | | binary_one_hot | | one_hot_dummy_na_true | | Yes |
| race_WHITE - BRAZILIAN | | binary_one_hot | | one_hot_dummy_na_true | | Yes |
| race_WHITE - EASTERN EUROPEAN | | binary_one_hot | | one_hot_dummy_na_true | | Yes |
| race_WHITE - OTHER EUROPEAN | | binary_one_hot | | one_hot_dummy_na_true | | Yes |
| race_WHITE - RUSSIAN | | binary_one_hot | | one_hot_dummy_na_true | | Yes |
| race_nan | | binary_one_hot | | one_hot_dummy_na_true | | Yes |
| insurance_Medicaid | | binary_one_hot | | one_hot_dummy_na_true | | Yes |
| insurance_Medicare | | binary_one_hot | | one_hot_dummy_na_true | | Yes |
| insurance_No charge | | binary_one_hot | | one_hot_dummy_na_true | | Yes |
| insurance_Other | | binary_one_hot | | one_hot_dummy_na_true | | Yes |
| insurance_Private | | binary_one_hot | | one_hot_dummy_na_true | | Yes |
| insurance_nan | | binary_one_hot | | one_hot_dummy_na_true | | Yes |
| language_American Sign Language | | binary_one_hot | | one_hot_dummy_na_true | | Yes |
| language_Amharic | | binary_one_hot | | one_hot_dummy_na_true | | Yes |
| language_Arabic | | binary_one_hot | | one_hot_dummy_na_true | | Yes |
| language_Armenian | | binary_one_hot | | one_hot_dummy_na_true | | Yes |
| language_Bengali | | binary_one_hot | | one_hot_dummy_na_true | | Yes |
| language_Chinese | | binary_one_hot | | one_hot_dummy_na_true | | Yes |
| language_English | | binary_one_hot | | one_hot_dummy_na_true | | Yes |
| language_French | | binary_one_hot | | one_hot_dummy_na_true | | Yes |
| language_Haitian | | binary_one_hot | | one_hot_dummy_na_true | | Yes |
| language_Hindi | | binary_one_hot | | one_hot_dummy_na_true | | Yes |
| language_Italian | | binary_one_hot | | one_hot_dummy_na_true | | Yes |
| language_Japanese | | binary_one_hot | | one_hot_dummy_na_true | | Yes |
| language_Kabuverdianu | | binary_one_hot | | one_hot_dummy_na_true | | Yes |
| language_Khmer | | binary_one_hot | | one_hot_dummy_na_true | | Yes |
| language_Korean | | binary_one_hot | | one_hot_dummy_na_true | | Yes |
| language_Modern Greek | | binary_one_hot | | one_hot_dummy_na_true | | Yes |
| language_Other | | binary_one_hot | | one_hot_dummy_na_true | | Yes |
| language_Persian | | binary_one_hot | | one_hot_dummy_na_true | | Yes |
| language_Polish | | binary_one_hot | | one_hot_dummy_na_true | | Yes |
| language_Portuguese | | binary_one_hot | | one_hot_dummy_na_true | | Yes |
| language_Russian | | binary_one_hot | | one_hot_dummy_na_true | | Yes |
| language_Somali | | binary_one_hot | | one_hot_dummy_na_true | | Yes |
| language_Spanish | | binary_one_hot | | one_hot_dummy_na_true | | Yes |
| language_Thai | | binary_one_hot | | one_hot_dummy_na_true | | Yes |
| language_Vietnamese | | binary_one_hot | | one_hot_dummy_na_true | | Yes |
| language_nan | | binary_one_hot | | one_hot_dummy_na_true | | Yes |
| marital_status_DIVORCED | | binary_one_hot | | one_hot_dummy_na_true | | Yes |
| marital_status_MARRIED | | binary_one_hot | | one_hot_dummy_na_true | | Yes |
| marital_status_SINGLE | | binary_one_hot | | one_hot_dummy_na_true | | Yes |
| marital_status_WIDOWED | | binary_one_hot | | one_hot_dummy_na_true | | Yes |
| marital_status_nan | | binary_one_hot | | one_hot_dummy_na_true | | Yes |
| micu_code_CCU | | binary_one_hot | | one_hot_dummy_na_true | | Yes |
| micu_code_CVICU | | binary_one_hot | | one_hot_dummy_na_true | | Yes |
| micu_code_ICU | | binary_one_hot | | one_hot_dummy_na_true | | Yes |
| micu_code_MICU | | binary_one_hot | | one_hot_dummy_na_true | | Yes |
| micu_code_MICU/SICU | | binary_one_hot | | one_hot_dummy_na_true | | Yes |
| micu_code_Med/Surg | | binary_one_hot | | one_hot_dummy_na_true | | Yes |
| micu_code_Medicine | | binary_one_hot | | one_hot_dummy_na_true | | Yes |
| micu_code_Medicine/Cardiology Intermediate | | binary_one_hot | | one_hot_dummy_na_true | | Yes |
| micu_code_Neuro Intermediate | | binary_one_hot | | one_hot_dummy_na_true | | Yes |
| micu_code_Neuro SICU | | binary_one_hot | | one_hot_dummy_na_true | | Yes |
| micu_code_Neuro Stepdown | | binary_one_hot | | one_hot_dummy_na_true | | Yes |
| micu_code_Neurology | | binary_one_hot | | one_hot_dummy_na_true | | Yes |
| micu_code_PACU | | binary_one_hot | | one_hot_dummy_na_true | | Yes |
| micu_code_SICU | | binary_one_hot | | one_hot_dummy_na_true | | Yes |
| micu_code_Surgery/Trauma | | binary_one_hot | | one_hot_dummy_na_true | | Yes |
| micu_code_Surgery/Vascular/Intermediate | | binary_one_hot | | one_hot_dummy_na_true | | Yes |
| micu_code_TSICU | | binary_one_hot | | one_hot_dummy_na_true | | Yes |
| micu_code_nan | binary_one_hot | | one_hot_dummy_na_true | | Yes | |
