# Supplementary material for: Development and Interpretability Analysis of a Stacking Ensemble Model for Early Prediction of Nutritional Risk in Intensive Care Unit Patients: Retrospective Cohort Study
Source: JMIR Med Inform. 2026 Jun 3;14:e77872. doi: 10.2196/77872 (PMC13232782; doi:10.2196/77872)
Supplement: Multimedia Appendix 2 [file medinform-v14-e77872-s002.docx]

Multimedia Appendix 2. Optimization of Data Preprocessing Strategies

To determine the most robust data preprocessing pipeline for the E-NUTRIC model, we conducted a series of preliminary experiments comparing different strategies for handling missing values and class imbalance. These evaluations were performed using a 5-fold cross-validation scheme on the training set to ensure unbiased selection. We first evaluated three imputation techniques: Mean Imputation, K-Nearest Neighbors (KNN), and Multiple Imputation by Chained Equations (MICE). As summarized in Table S2, the KNN imputation method yielded the highest discriminative performance with a mean ROC-AUC of 0.8611 (SD ± 0.0064), marginally outperforming Mean Imputation (0.8610) and significantly surpassing MICE (0.8519). Based on these results, KNN was adopted for the final model. Subsequently, to address the dataset’s class imbalance (8.5% positive prevalence), we compared Synthetic Minority Over-sampling Technique (SMOTE), Random Under-Sampling (RUS), and Class Weighting. The Area Under the Precision-Recall Curve (AUPRC) was utilized as the decision metric. Random Under-Sampling achieved the highest mean AUPRC of 0.3269, demonstrating a superior trade-off between precision and recall compared to SMOTE (0.3250) and Class Weighting (0.3241), leading to its inclusion in the final methodology.

Table S1. Comparative performance of data preprocessing strategies.

| Experiment | Pipeline | Metric | Mean Score | Standard Deviation |
| --- | --- | --- | --- | --- |
| Imputation Strategy | Pipe_KNN (Selected) | ROC-AUC | 0.8611 | 0.0064 |
|  | Pipe_Mean | ROC-AUC | 0.861 | 0.0085 |
|  | Pipe_MICE | ROC-AUC | 0.8519 | 0.0056 |
| Resampling Strategy | Pipe_Under (Selected) | AUPRC | 0.3269 | 0.0182 |
|  | Pipe_SMOTE | AUPRC | 0.325 | 0.0072 |
|  | Pipe_Weight | AUPRC | 0.3241 | 0.0058 |
