# Supplementary material for: Development and Interpretability Analysis of a Stacking Ensemble Model for Early Prediction of Nutritional Risk in Intensive Care Unit Patients: Retrospective Cohort Study
Source: JMIR Med Inform. 2026 Jun 3;14:e77872. doi: 10.2196/77872 (PMC13232782; doi:10.2196/77872)
Supplement: Multimedia Appendix 3 [file medinform-v14-e77872-s003.docx]

Multimedia Appendix 3. Hyperparameter Settings and Model Configuration

This appendix provides the detailed configuration and hyperparameter settings for the data preprocessing pipeline, base learners (Level-0), and the stacking meta-learner (Level-1) used in this study. To ensure reproducibility and prevent data leakage, all models were trained using a fixed random seed (random_state=42 where applicable). To prevent over-prediction and model distortion, algorithm-level class weighting hyperparameters were explicitly disabled across all learners, relying exclusively on Random Under-Sampling within the pipeline to handle the baseline class imbalance.

## Data Preprocessing and Imputation

Before model training, the dataset underwent a unified preprocessing pipeline. The specific configurations for imputation and resampling are listed below.

Table S1.

| Component | Method | Hyperparameters / Configuration |
| --- | --- | --- |
| Missing Value Imputation | KNN Imputer | n_neighbors: 5 |
|  |  | weights: ‘uniform’ |
|  |  | metric: ‘nan_euclidean’ |
| Feature Scaling | Standard Scaler | with_mean: True |
|  |  | with_std: True |
| Class Balancing | Random Under-Sampling | sampling_strategy: ‘auto’ (targets a 1:1 ratio) |
|  |  | replacement: False |

## Level-0 Base Learners

Four distinct machine learning algorithms were employed as base learners in the stacking ensemble. The hyperparameters were pre-selected based on empirical performance and fixed for the final training phase to ensure robustness.

1. Logistic Regression (LR)

Used as a linear baseline and for its interpretability.

Table S2.

| Parameter | Value | Description |
| --- | --- | --- |
| penalty | ‘l2’ | Ridge regularization |
| C | 0.1 | Inverse of regularization strength |
| solver | ‘saga’ | Algorithm for optimization (supports L1/L2) |
| class_weight | None | Explicitly disabled; balance is handled by Random Under-Sampling |
| max_iter | 1000 | Maximum number of iterations for solver convergence |

2. Random Forest (RF)

An ensemble of decision trees using bagging.

Table S3.

| Parameter | Value | Description |
| --- | --- | --- |
| n_estimators | 500 | Number of trees in the forest |
| criterion | ‘gini’ | Function to measure the quality of a split |
| class_weight | None | Explicitly disabled to prevent distortion post-resampling |
| bootstrap | TRUE | Whether bootstrap samples are used when building trees |

3. LightGBM

A gradient boosting framework that uses tree-based learning algorithms.

Table S4.

| Parameter | Value | Description |
| --- | --- | --- |
| n_estimators | 500 | Number of boosting iterations |
| learning_rate | 0.05 | Boosting learning rate |
| num_leaves | 31 | Maximum tree leaves for base learners |
| objective | ‘binary’ | Specify the learning task |
| is_unbalance | False | Disabled to maintain neutral weighting on the balanced pipeline |

4. XGBoost

An optimized distributed gradient boosting library.

Table S5.

| Parameter | Value | Description |
| --- | --- | --- |
| n_estimators | 500 | Number of gradient boosted trees |
| learning_rate | 0.05 | Step size shrinkage used in update to prevents overfitting |
| max_depth | 6 | Maximum depth of a tree |
| subsample | 0.8 | Subsample ratio of the training instances |
| colsample_bytree | 0.8 | Subsample ratio of columns when constructing each tree |
| scale_pos_weight | 1 | Set to neutral (1) to prevent severe over-prediction of the minority class |
| objective | ‘binary:logistic’ | Logistic regression for binary classification |

## Level-1 Stacking Meta-Learner

The outputs (predicted probabilities) of the four base learners were used as input features for the meta-learner.

Table S6.

| Model | Hyperparameters |
| --- | --- |
| Logistic Regression | C: 0.1 |
|  | solver: ‘saga’ |
|  | penalty: ‘l2’ |
|  | max_iter: 1000 |
|  | class_weight: None |
| Stacking Strategy | 5-Fold Cross-Validation |
|  | The meta-learner was trained on out-of-fold predictions from the base learners to prevent overfitting. |

## Software Environment

The models were implemented using Python 3.10 with the following primary libraries:

Scikit-learn: v1.7.2 (Logistic Regression, Random Forest, KNN Imputer, StackingClassifier)

XGBoost: v3.1.2

LightGBM: v4.6.0

Imbalanced-learn: v0.14.1 (RandomUnderSampler)
