# Supplementary material for: Development and Interpretability Analysis of a Stacking Ensemble Model for Early Prediction of Nutritional Risk in Intensive Care Unit Patients: Retrospective Cohort Study
Source: JMIR Med Inform. 2026 Jun 3;14:e77872. doi: 10.2196/77872 (PMC13232782; doi:10.2196/77872)
Supplement: Multimedia Appendix 4 [file medinform-v14-e77872-s004.docx]

Multimedia Appendix 4. Comprehensive Baseline Characteristics and Feature Statistics

Table S1. Comprehensive Baseline Statistical Comparison of All Candidate Features

This table presents the exhaustive statistical comparison of all candidate predictors extracted during the initial 24-hour ICU stay between the Malnutrition Risk Group and the No Risk Group. Data Presentation and Statistical Tests: Validating the long-tail nature of clinical data, all continuous variables are expressed uniformly as median with interquartile range [IQR] and compared using the Mann-Whitney U test. Categorical variables are presented as counts and percentages (n, %) and compared using the Chi-square test. Variable Standardization: To ensure readability, raw electronic health record (EHR) variable codes and item IDs have been systematically mapped to standard medical terminology. Time-series physiological and laboratory parameters are aggregated into six statistical metrics (Mean, Min, Max, Standard Deviation [Std], First, and Last) within the 24-hour presentation window. Data Preprocessing Note (Feature Count Discrepancy): Readers may note a numerical difference between the 297 functional features used in the final machine learning predictive models (detailed in Multimedia Appendix 1) and the 265 variables presented in this clinical baseline table. This discrepancy is a standard artifact of the machine learning preprocessing pipeline. The 297-column algorithmic matrix includes computationally necessary but clinically redundant operational columns. Specifically, it includes extended one-hot encoded “missing value indicators” (e.g., explicitly encoded_nan columns for missing gender or insurance representations) and dynamically split highly sparse sub-categories. Furthermore, 10 static admission vital sign summaries (e.g., Vitalhr or SpO2) that topographically overlapped with the computed 24-hour time-series aggregations were omitted from this descriptive table to prevent clinical redundancy and improve readability. Because these operational columns are mathematically necessary for the algorithm but inappropriate for standard descriptive clinical statistics, they have been appropriately consolidated or omitted in this baseline table.

Abbreviations: IQR: Interquartile Range; ICU: Intensive Care Unit; NIBP: Non-Invasive Blood Pressure; ABP: Arterial Blood Pressure; SpO2: Peripheral Capillary Oxygen Saturation; BUN: Blood Urea Nitrogen.

| Variable | | | Risk Group (N=4384) | No Risk Group (N=47099) | *P* value | Test Method |
| --- | --- | --- | --- | --- | --- | --- |
| Demographics & Socioeconomics | | |  |  |  |  |
|  | Age (years), median [IQR] | | 68.00 [57.00, 78.00] | 67.00 [56.00, 78.00] | .002 | Mann-Whitney U |
|  | **Gender, n (%)** | |  |  | .02 | Chi-square |
|  |  | Female | 1948 (44.4) | 20069 (42.6) |  |  |
|  |  | Male | 2436 (55.6) | 27030 (57.4) |  |  |
|  | **Race Enc, n (%)** | |  |  | <.001 | Chi-square |
|  |  | AMERICAN INDIAN/ALASKA NATIVE | 12 (0.3) | 87 (0.2) |  |  |
|  |  | ASIAN | 49 (1.1) | 557 (1.2) |  |  |
|  |  | ASIAN - ASIAN INDIAN | 12 (0.3) | 124 (0.3) |  |  |
|  |  | ASIAN - CHINESE | 59 (1.3) | 488 (1.0) |  |  |
|  |  | ASIAN - KOREAN | 3 (0.1) | 46 (0.1) |  |  |
|  |  | ASIAN - SOUTH EAST ASIAN | 24 (0.5) | 150 (0.3) |  |  |
|  |  | BLACK/AFRICAN | 20 (0.5) | 196 (0.4) |  |  |
|  |  | BLACK/AFRICAN AMERICAN | 367 (8.4) | 3332 (7.1) |  |  |
|  |  | BLACK/CAPE VERDEAN | 27 (0.6) | 271 (0.6) |  |  |
|  |  | BLACK/CARIBBEAN ISLAND | 36 (0.8) | 274 (0.6) |  |  |
|  |  | HISPANIC OR LATINO | 10 (0.2) | 392 (0.8) |  |  |
|  |  | HISPANIC/LATINO - CENTRAL AMERICAN | 5 (0.1) | 28 (0.1) |  |  |
|  |  | HISPANIC/LATINO - COLUMBIAN | 2 (0.0) | 42 (0.1) |  |  |
|  |  | HISPANIC/LATINO - CUBAN | 2 (0.0) | 40 (0.1) |  |  |
|  |  | HISPANIC/LATINO - DOMINICAN | 20 (0.5) | 384 (0.8) |  |  |
|  |  | HISPANIC/LATINO - GUATEMALAN | 10 (0.2) | 93 (0.2) |  |  |
|  |  | HISPANIC/LATINO - HONDURAN | 5 (0.1) | 38 (0.1) |  |  |
|  |  | HISPANIC/LATINO - MEXICAN | 3 (0.1) | 52 (0.1) |  |  |
|  |  | HISPANIC/LATINO - PUERTO RICAN | 68 (1.6) | 500 (1.1) |  |  |
|  |  | HISPANIC/LATINO - SALVADORAN | 10 (0.2) | 84 (0.2) |  |  |
|  |  | MULTIPLE RACE/ETHNICITY | 3 (0.1) | 37 (0.1) |  |  |
|  |  | NATIVE HAWAIIAN OR OTHER PACIFIC ISLANDER | 6 (0.1) | 66 (0.1) |  |  |
|  |  | OTHER | 140 (3.2) | 1651 (3.5) |  |  |
|  |  | PATIENT DECLINED TO ANSWER | 15 (0.3) | 360 (0.8) |  |  |
|  |  | PORTUGUESE | 21 (0.5) | 152 (0.3) |  |  |
|  |  | SOUTH AMERICAN | 7 (0.2) | 41 (0.1) |  |  |
|  |  | UNABLE TO OBTAIN | 96 (2.2) | 1267 (2.7) |  |  |
|  |  | UNKNOWN | 551 (12.6) | 5373 (11.4) |  |  |
|  |  | WHITE | 2642 (60.3) | 29197 (62.0) |  |  |
|  |  | WHITE - BRAZILIAN | 8 (0.2) | 104 (0.2) |  |  |
|  |  | WHITE - EASTERN EUROPEAN | 12 (0.3) | 135 (0.3) |  |  |
|  |  | WHITE - OTHER EUROPEAN | 106 (2.4) | 1148 (2.4) |  |  |
|  |  | WHITE - RUSSIAN | 33 (0.8) | 390 (0.8) |  |  |
|  | **Marital Status Enc, n (%)** | |  |  | <.001 | Chi-square |
|  |  | DIVORCED | 350 (8.0) | 3247 (6.9) |  |  |
|  |  | MARRIED | 1710 (39.0) | 21603 (45.9) |  |  |
|  |  | NULL | 545 (12.4) | 4812 (10.2) |  |  |
|  |  | SINGLE | 1295 (29.5) | 11999 (25.5) |  |  |
|  |  | WIDOWED | 484 (11.0) | 5438 (11.5) |  |  |
|  | **Insurance Enc, n (%)** | |  |  | <.001 | Chi-square |
|  |  | Medicaid | 754 (17.2) | 6376 (13.5) |  |  |
|  |  | Medicare | 2447 (55.8) | 25236 (53.6) |  |  |
|  |  | NULL | 51 (1.2) | 831 (1.8) |  |  |
|  |  | Other | 84 (1.9) | 1274 (2.7) |  |  |
|  |  | Private | 1048 (23.9) | 13376 (28.4) |  |  |
|  | **Language Enc, n (%)** | |  |  | <.001 | Chi-square |
|  |  | American Sign Language | 4 (0.1) | 30 (0.1) |  |  |
|  |  | Amharic | 1 (0.0) | 17 (0.0) |  |  |
|  |  | Arabic | 13 (0.3) | 76 (0.2) |  |  |
|  |  | Armenian | 1 (0.0) | 12 (0.0) |  |  |
|  |  | Bengali | 1 (0.0) | 10 (0.0) |  |  |
|  |  | Chinese | 74 (1.7) | 554 (1.2) |  |  |
|  |  | English | 3917 (89.3) | 42564 (90.4) |  |  |
|  |  | French | 2 (0.0) | 17 (0.0) |  |  |
|  |  | Haitian | 48 (1.1) | 299 (0.6) |  |  |
|  |  | Hindi | 4 (0.1) | 26 (0.1) |  |  |
|  |  | Italian | 9 (0.2) | 100 (0.2) |  |  |
|  |  | Japanese | 0 (0.0) | 5 (0.0) |  |  |
|  |  | Kabuverdianu | 29 (0.7) | 283 (0.6) |  |  |
|  |  | Khmer | 7 (0.2) | 36 (0.1) |  |  |
|  |  | Korean | 1 (0.0) | 41 (0.1) |  |  |
|  |  | Modern Greek | 9 (0.2) | 84 (0.2) |  |  |
|  |  | NULL | 16 (0.4) | 264 (0.6) |  |  |
|  |  | Other | 20 (0.5) | 140 (0.3) |  |  |
|  |  | Persian | 2 (0.0) | 39 (0.1) |  |  |
|  |  | Polish | 3 (0.1) | 32 (0.1) |  |  |
|  |  | Portuguese | 36 (0.8) | 336 (0.7) |  |  |
|  |  | Russian | 37 (0.8) | 550 (1.2) |  |  |
|  |  | Somali | 1 (0.0) | 5 (0.0) |  |  |
|  |  | Spanish | 124 (2.8) | 1436 (3.0) |  |  |
|  |  | Thai | 2 (0.0) | 19 (0.0) |  |  |
|  |  | Vietnamese | 23 (0.5) | 124 (0.3) |  |  |
| Anthropometrics | | |  |  |  |  |
|  |  | BMI, median [IQR] | 25.19 [20.99, 30.06] | 28.34 [24.76, 32.89] | <.001 | Mann-Whitney U |
|  |  | Weight, median [IQR] | 69.53 [57.30, 85.00] | 80.30 [68.00, 95.35] | <.001 | Mann-Whitney U |
|  |  | Height, median [IQR] | 168.00 [161.00, 178.00] | 170.00 [163.00, 178.00] | <.001 | Mann-Whitney U |
|  |  | Unintentional Weight Loss, n (%) | 593 (13.5) | 2005 (4.3) | <.001 | Chi-square |
|  |  | Lab 24Hour Dailyweight, median [IQR] | 70.70 [58.45, 86.70] | 83.90 [70.30, 99.00] | <.001 | Mann-Whitney U |
| ICU Admission & Clinical Scores | | |  |  |  |  |
|  | **ICU Unit Types, n (%)** | |  |  | <.001 | Chi-square |
|  |  | CCU | 324 (7.4) | 5404 (11.5) |  |  |
|  |  | CVICU | 208 (4.7) | 10418 (22.1) |  |  |
|  |  | MICU | 1260 (28.7) | 7990 (17.0) |  |  |
|  |  | MICU/SICU | 1088 (24.8) | 6164 (13.1) |  |  |
|  |  | Neuro Intermediate | 168 (3.8) | 3462 (7.4) |  |  |
|  |  | Neuro SICU | 88 (2.0) | 1002 (2.1) |  |  |
|  |  | Neuro Stepdown | 38 (0.9) | 791 (1.7) |  |  |
|  |  | Other | 47 (1.1) | 141 (0.3) |  |  |
|  |  | SICU | 705 (16.1) | 6361 (13.5) |  |  |
|  |  | TSICU | 458 (10.4) | 5366 (11.4) |  |  |
|  | Pre-ICU LoS, median [IQR] | | 0.09 [0.04, 2.14] | 0.08 [0.03, 0.75] | <.001 | Mann-Whitney U |
|  | APACHE II Score, median [IQR] | | 22.00 [17.00, 27.00] | 18.00 [13.00, 23.00] | <.001 | Mann-Whitney U |
|  | Charlson Comorbidity Index, median [IQR] | | 6.00 [4.00, 8.00] | 5.00 [3.00, 7.00] | <.001 | Mann-Whitney U |
|  | mNUTRIC Score, median [IQR] | | 5.00 [4.00, 6.00] | 4.00 [2.00, 5.00] | <.001 | Mann-Whitney U |
|  | Acute Physiology Score, median [IQR] | | 15.00 [11.00, 19.00] | 11.00 [8.00, 16.00] | <.001 | Mann-Whitney U |
|  | Age Score, median [IQR] | | 5.00 [3.00, 6.00] | 5.00 [3.00, 6.00] | .007 | Mann-Whitney U |
|  | Chronic Score, median [IQR] | | 5.00 [0.00, 5.00] | 0.00 [0.00, 5.00] | <.001 | Mann-Whitney U |
|  | SOFA Score, median [IQR] | | 5.00 [3.00, 8.00] | 4.00 [2.00, 6.00] | <.001 | Mann-Whitney U |
|  | APS III, median [IQR] | | 50.00 [38.00, 65.00] | 38.00 [28.00, 51.00] | <.001 | Mann-Whitney U |
|  | SIRS, median [IQR] | | 3.00 [2.00, 3.00] | 3.00 [2.00, 3.00] | <.001 | Mann-Whitney U |
|  | SAPS II, median [IQR] | | 40.00 [32.00, 50.00] | 34.00 [26.00, 43.00] | <.001 | Mann-Whitney U |
|  | OASIS, median [IQR] | | 34.00 [28.00, 40.00] | 30.00 [25.00, 36.00] | <.001 | Mann-Whitney U |
|  | GCS, median [IQR] | | 15.00 [13.00, 15.00] | 15.00 [14.00, 15.00] | <.001 | Mann-Whitney U |
| Interventions, Treatments & Nutrition | | |  |  |  |  |
|  | Norepinephrine, n (%) | | 1150 (26.2) | 6335 (13.5) | <.001 | Chi-square |
|  | Epinephrine, n (%) | | 85 (1.9) | 1997 (4.2) | <.001 | Chi-square |
|  | Vasopressin, n (%) | | 313 (7.1) | 1701 (3.6) | <.001 | Chi-square |
|  | Enteral Nutrition, n (%) | | 101 (2.3) | 334 (0.7) | <.001 | Chi-square |
|  | Parenteral Nutrition, n (%) | | 124 (2.8) | 141 (0.3) | <.001 | Chi-square |
|  | Special Components, n (%) | | 810 (18.5) | 7924 (16.8) | .005 | Chi-square |
|  | Glucocorticoids, n (%) | | 209 (4.8) | 1998 (4.2) | .101 | Chi-square |
|  | Metoclopramide, n (%) | | 28 (0.6) | 442 (0.9) | .046 | Chi-square |
|  | Supplements, n (%) | | 257 (5.9) | 1256 (2.7) | <.001 | Chi-square |
|  | Insulin, n (%) | | 619 (14.1) | 7034 (14.9) | .147 | Chi-square |
|  | Hormone, n (%) | | 114 (2.6) | 950 (2.0) | .009 | Chi-square |
|  | Gastrostomy, n (%) | | 346 (7.9) | 712 (1.5) | <.001 | Chi-square |
|  | Ventilation, n (%) | | 2020 (46.1) | 12239 (26.0) | <.001 | Chi-square |
|  | Dialysis, n (%) | | 143 (3.3) | 1268 (2.7) | .027 | Chi-square |
|  | Bypass, n (%) | | 232 (5.3) | 5645 (12.0) | <.001 | Chi-square |
| Vital Signs (24h Aggregations), median [IQR] | | |  |  |  |  |
|  | Diastolic Bp (Mean) | | 62.34 [56.00, 70.17] | 62.50 [56.00, 70.50] | .437 | Mann-Whitney U |
|  | Heart Rate (Mean) | | 87.85 [76.68, 100.44] | 82.35 [73.14, 93.35] | <.001 | Mann-Whitney U |
|  | Mean Bp (Mean) | | 76.46 [70.29, 84.43] | 77.77 [71.66, 85.81] | <.001 | Mann-Whitney U |
|  | Respiratory Rate (Mean) | | 19.35 [16.75, 22.46] | 18.32 [16.35, 20.88] | <.001 | Mann-Whitney U |
|  | SpO2 (Mean) | | 97.08 [95.60, 98.54] | 97.09 [95.72, 98.38] | .51 | Mann-Whitney U |
|  | Systolic Bp (Mean) | | 112.24 [102.89, 124.88] | 116.48 [107.44, 128.42] | <.001 | Mann-Whitney U |
|  | Temperature (Mean) | | 98.27 [97.90, 98.77] | 98.30 [97.92, 98.75] | .098 | Mann-Whitney U |
|  | Diastolic Bp (Std) | | 9.16 [6.98, 12.01] | 9.02 [6.94, 11.82] | .07 | Mann-Whitney U |
|  | Heart Rate (Std) | | 8.22 [6.00, 11.26] | 7.91 [5.90, 10.67] | <.001 | Mann-Whitney U |
|  | Mean Bp (Std) | | 9.84 [7.41, 13.05] | 9.83 [7.68, 12.82] | .331 | Mann-Whitney U |
|  | Respiratory Rate (Std) | | 3.82 [2.93, 4.88] | 3.67 [2.89, 4.66] | <.001 | Mann-Whitney U |
|  | SpO2 (Std) | | 1.89 [1.34, 2.54] | 1.82 [1.35, 2.38] | <.001 | Mann-Whitney U |
|  | Systolic Bp (Std) | | 13.28 [10.12, 16.95] | 13.17 [10.46, 16.62] | .867 | Mann-Whitney U |
|  | Temperature (Std) | | 0.52 [0.34, 0.83] | 0.52 [0.34, 0.82] | .939 | Mann-Whitney U |
|  | Diastolic Bp (First) | | 67.00 [57.00, 80.00] | 67.00 [57.00, 79.00] | .176 | Mann-Whitney U |
|  | Heart Rate (First) | | 92.00 [79.00, 109.00] | 84.00 [74.00, 98.00] | <.001 | Mann-Whitney U |
|  | Mean Bp (First) | | 82.00 [70.00, 95.00] | 83.00 [72.00, 95.00] | <.001 | Mann-Whitney U |
|  | Respiratory Rate (First) | | 20.00 [16.00, 24.00] | 18.00 [15.00, 22.00] | <.001 | Mann-Whitney U |
|  | SpO2 (First) | | 98.00 [95.00, 100.00] | 98.00 [96.00, 100.00] | <.001 | Mann-Whitney U |
|  | Systolic Bp (First) | | 119.00 [103.00, 137.00] | 123.00 [108.00, 140.00] | <.001 | Mann-Whitney U |
|  | Temperature (First) | | 98.20 [97.65, 98.80] | 98.10 [97.60, 98.70] | .003 | Mann-Whitney U |
|  | Diastolic Bp (Last) | | 61.00 [53.00, 71.00] | 62.00 [53.00, 72.00] | .021 | Mann-Whitney U |
|  | Heart Rate (Last) | | 86.00 [74.00, 99.00] | 82.00 [71.00, 94.00] | <.001 | Mann-Whitney U |
|  | Mean Bp (Last) | | 76.00 [68.00, 87.00] | 77.00 [69.00, 88.00] | <.001 | Mann-Whitney U |
|  | Respiratory Rate (Last) | | 19.00 [16.00, 23.00] | 19.00 [16.00, 22.00] | <.001 | Mann-Whitney U |
|  | SpO2 (Last) | | 97.00 [95.00, 99.00] | 97.00 [95.00, 99.00] | <.001 | Mann-Whitney U |
|  | Systolic Bp (Last) | | 113.00 [101.00, 129.00] | 117.00 [105.00, 132.00] | <.001 | Mann-Whitney U |
|  | Temperature (Last) | | 98.30 [97.80, 98.90] | 98.40 [97.90, 98.90] | <.001 | Mann-Whitney U |
|  | Diastolic Bp (Min) | | 46.00 [40.00, 53.00] | 46.00 [40.00, 54.00] | .128 | Mann-Whitney U |
|  | Heart Rate (Min) | | 73.00 [62.00, 84.00] | 68.00 [59.00, 78.00] | <.001 | Mann-Whitney U |
|  | Mean Bp (Min) | | 59.00 [52.00, 66.00] | 60.00 [53.00, 68.00] | <.001 | Mann-Whitney U |
|  | Respiratory Rate (Min) | | 13.00 [10.00, 15.00] | 12.00 [10.00, 14.00] | <.001 | Mann-Whitney U |
|  | SpO2 (Min) | | 92.00 [90.00, 95.00] | 93.00 [90.00, 95.00] | <.001 | Mann-Whitney U |
|  | Systolic Bp (Min) | | 87.00 [78.00, 97.00] | 91.00 [82.00, 102.00] | <.001 | Mann-Whitney U |
|  | Temperature (Min) | | 97.60 [97.30, 98.00] | 97.70 [97.30, 98.10] | .001 | Mann-Whitney U |
|  | Diastolic Bp (Max) | | 87.00 [75.00, 101.00] | 86.00 [75.00, 99.00] | .006 | Mann-Whitney U |
|  | Heart Rate (Max) | | 107.00 [94.00, 122.00] | 100.00 [88.00, 114.00] | <.001 | Mann-Whitney U |
|  | Mean Bp (Max) | | 101.00 [90.00, 116.00] | 102.00 [92.00, 115.00] | .003 | Mann-Whitney U |
|  | Respiratory Rate (Max) | | 28.00 [24.00, 33.00] | 27.00 [23.00, 31.00] | <.001 | Mann-Whitney U |
|  | SpO2 (Max) | | 100.00 [100.00, 100.00] | 100.00 [99.00, 100.00] | .001 | Mann-Whitney U |
|  | Systolic Bp (Max) | | 143.00 [128.00, 160.00] | 146.00 [133.00, 161.00] | <.001 | Mann-Whitney U |
|  | Temperature (Max) | | 98.90 [98.50, 99.80] | 98.90 [98.50, 99.70] | .377 | Mann-Whitney U |
| Laboratory Values (24h Aggregations), median [IQR] | | |  |  |  |  |
|  | Albumin (Min) | | 2.40 [2.10, 2.90] | 3.00 [2.50, 3.40] | <.001 | Mann-Whitney U |
|  | Potassium (Min) | | 3.30 [3.00, 3.60] | 3.60 [3.30, 3.90] | <.001 | Mann-Whitney U |
|  | Potassium (Max) | | 5.00 [4.50, 5.60] | 4.60 [4.30, 5.10] | <.001 | Mann-Whitney U |
|  | Glucose (Min) | | 82.00 [70.00, 94.00] | 93.00 [82.00, 105.00] | <.001 | Mann-Whitney U |
|  | Creatinine (Min) | | 0.60 [0.40, 1.00] | 0.80 [0.60, 1.00] | <.001 | Mann-Whitney U |
|  | Creatinine (Mean) | | 0.91 [0.62, 1.55] | 0.90 [0.70, 1.27] | .324 | Mann-Whitney U |
|  | Lymphocytes (Absolute) (Min) | | 0.68 [0.36, 1.12] | 1.21 [0.70, 1.90] | <.001 | Mann-Whitney U |
|  | Phosphate (Min) | | 2.10 [1.70, 2.60] | 2.50 [2.00, 3.00] | <.001 | Mann-Whitney U |
|  | Phosphate (Max) | | 4.80 [4.00, 6.20] | 4.10 [3.50, 4.90] | <.001 | Mann-Whitney U |
|  | BUN (Std) | | 6.66 [3.66, 12.94] | 3.85 [2.28, 7.00] | <.001 | Mann-Whitney U |
|  | BUN (Max) | | 38.00 [23.00, 66.00] | 24.00 [17.00, 39.00] | <.001 | Mann-Whitney U |
|  | Magnesium (Max) | | 2.50 [2.30, 2.80] | 2.30 [2.10, 2.60] | <.001 | Mann-Whitney U |
|  | Glucose (Blood Gas) (Mean) | | 134.14 [113.67, 164.56] | 137.00 [121.40, 157.67] | <.001 | Mann-Whitney U |
|  | Lactate (Mean) | | 1.73 [1.25, 2.43] | 1.73 [1.30, 2.36] | .656 | Mann-Whitney U |
|  | Potassium (Blood Gas) (Mean) | | 3.97 [3.60, 4.40] | 4.19 [3.82, 4.54] | <.001 | Mann-Whitney U |
|  | Albumin (Mean) | | 2.74 [2.37, 3.14] | 3.10 [2.70, 3.50] | <.001 | Mann-Whitney U |
|  | Protein (Chemistry) (Mean) | | 85.40 [34.70, 143.38] | 67.20 [17.90, 130.42] | <.001 | Mann-Whitney U |
|  | Cholesterol (Mean) | | 117.00 [86.00, 152.75] | 150.00 [119.00, 185.00] | <.001 | Mann-Whitney U |
|  | Glucose (Chemistry) (Mean) | | 124.83 [108.51, 152.05] | 120.50 [106.75, 142.76] | <.001 | Mann-Whitney U |
|  | Magnesium (Mean) | | 2.03 [1.92, 2.17] | 2.05 [1.93, 2.20] | <.001 | Mann-Whitney U |
|  | Phosphate (Mean) | | 3.37 [2.95, 3.91] | 3.30 [2.87, 3.77] | <.001 | Mann-Whitney U |
|  | Potassium (Chemistry) (Mean) | | 4.06 [3.85, 4.30] | 4.08 [3.85, 4.32] | .015 | Mann-Whitney U |
|  | Total Protein (Chemistry) (Mean) | | 5.40 [4.80, 6.00] | 5.80 [5.10, 6.40] | <.001 | Mann-Whitney U |
|  | Transferrin (Mean) | | 130.00 [100.25, 167.00] | 162.00 [128.00, 202.00] | <.001 | Mann-Whitney U |
|  | Triglycerides (Mean) | | 133.00 [89.00, 204.30] | 125.00 [86.00, 198.00] | .005 | Mann-Whitney U |
|  | BUN (Mean) | | 23.55 [14.75, 39.03] | 18.25 [12.93, 28.00] | <.001 | Mann-Whitney U |
|  | Lymphocytes (Absolute) (Mean) | | 1.02 [0.64, 1.52] | 1.40 [0.89, 2.04] | <.001 | Mann-Whitney U |
|  | Lymphocytes (%) (Mean) | | 9.60 [5.95, 15.00] | 12.30 [7.67, 18.40] | <.001 | Mann-Whitney U |
|  | Erythrocyte Sedimentation Rate (Mean) | | 67.00 [33.00, 95.00] | 55.00 [25.00, 90.00] | .058 | Mann-Whitney U |
|  | Glucose (Blood Gas) (Std) | | 28.65 [16.85, 45.47] | 24.84 [16.44, 36.60] | <.001 | Mann-Whitney U |
|  | Lactate (Std) | | 0.58 [0.31, 1.07] | 0.56 [0.30, 0.99] | <.001 | Mann-Whitney U |
|  | Potassium (Blood Gas) (Std) | | 0.42 [0.26, 0.63] | 0.46 [0.31, 0.64] | <.001 | Mann-Whitney U |
|  | Albumin (Std) | | 0.26 [0.15, 0.39] | 0.21 [0.14, 0.35] | <.001 | Mann-Whitney U |
|  | Protein (Chemistry) (Std) | | 36.01 [16.36, 66.17] | 42.91 [15.91, 74.53] | .144 | Mann-Whitney U |
|  | Cholesterol (Std) | | 9.19 [3.20, 26.32] | 9.19 [3.54, 20.51] | .455 | Mann-Whitney U |
|  | Creatinine (Std) | | 0.18 [0.10, 0.43] | 0.11 [0.07, 0.21] | <.001 | Mann-Whitney U |
|  | Glucose (Chemistry) (Std) | | 28.51 [19.14, 48.52] | 22.20 [14.05, 36.86] | <.001 | Mann-Whitney U |
|  | Magnesium (Std) | | 0.23 [0.17, 0.31] | 0.20 [0.13, 0.28] | <.001 | Mann-Whitney U |
|  | Phosphate (Std) | | 0.76 [0.55, 1.06] | 0.63 [0.42, 0.87] | <.001 | Mann-Whitney U |
|  | Potassium (Chemistry) (Std) | | 0.44 [0.34, 0.57] | 0.36 [0.26, 0.48] | <.001 | Mann-Whitney U |
|  | Total Protein (Chemistry) (Std) | | 0.28 [0.14, 0.55] | 0.28 [0.14, 0.51] | .574 | Mann-Whitney U |
|  | Transferrin (Std) | | 14.14 [6.83, 28.11] | 12.73 [5.51, 27.58] | .374 | Mann-Whitney U |
|  | Triglycerides (Std) | | 45.58 [17.65, 101.82] | 50.21 [18.72, 115.86] | .116 | Mann-Whitney U |
|  | Lymphocytes (Absolute) (Std) | | 0.33 [0.17, 0.59] | 0.35 [0.17, 0.61] | .333 | Mann-Whitney U |
|  | Lymphocytes (%) (Std) | | 3.49 [1.98, 6.09] | 3.54 [1.84, 6.18] | .787 | Mann-Whitney U |
|  | Erythrocyte Sedimentation Rate (Std) | | 12.53 [9.12, 40.33] | 10.61 [3.77, 20.15] | .029 | Mann-Whitney U |
|  | Glucose (Blood Gas) (First) | | 132.00 [108.00, 167.00] | 134.00 [112.00, 162.00] | .089 | Mann-Whitney U |
|  | Lactate (First) | | 1.60 [1.10, 2.30] | 1.60 [1.10, 2.30] | .035 | Mann-Whitney U |
|  | Potassium (Blood Gas) (First) | | 3.90 [3.60, 4.50] | 4.10 [3.70, 4.60] | <.001 | Mann-Whitney U |
|  | Albumin (First) | | 2.70 [2.30, 3.20] | 3.10 [2.70, 3.50] | <.001 | Mann-Whitney U |
|  | Protein (Chemistry) (First) | | 81.10 [27.65, 148.55] | 62.20 [14.70, 135.10] | <.001 | Mann-Whitney U |
|  | Cholesterol (First) | | 118.00 [86.00, 152.00] | 150.00 [119.00, 185.00] | <.001 | Mann-Whitney U |
|  | Creatinine (First) | | 0.90 [0.60, 1.50] | 0.90 [0.70, 1.30] | <.001 | Mann-Whitney U |
|  | Glucose (Chemistry) (First) | | 120.00 [99.00, 150.00] | 117.00 [99.00, 144.00] | .004 | Mann-Whitney U |
|  | Magnesium (First) | | 2.00 [1.80, 2.20] | 2.00 [1.90, 2.20] | <.001 | Mann-Whitney U |
|  | Phosphate (First) | | 3.30 [2.70, 4.10] | 3.30 [2.70, 3.90] | <.001 | Mann-Whitney U |
|  | Potassium (Chemistry) (First) | | 4.00 [3.70, 4.40] | 4.10 [3.70, 4.40] | <.001 | Mann-Whitney U |
|  | Total Protein (Chemistry) (First) | | 5.40 [4.80, 6.00] | 5.80 [5.10, 6.40] | <.001 | Mann-Whitney U |
|  | Transferrin (First) | | 130.50 [99.00, 168.00] | 162.00 [128.00, 203.00] | <.001 | Mann-Whitney U |
|  | Triglycerides (First) | | 130.00 [85.00, 197.75] | 123.00 [85.00, 193.00] | .155 | Mann-Whitney U |
|  | BUN (First) | | 22.00 [13.00, 38.00] | 18.00 [12.00, 28.00] | <.001 | Mann-Whitney U |
|  | Lymphocytes (Absolute) (First) | | 0.96 [0.58, 1.53] | 1.38 [0.86, 2.06] | <.001 | Mann-Whitney U |
|  | Lymphocytes (%) (First) | | 9.00 [5.00, 14.80] | 12.20 [7.20, 18.80] | <.001 | Mann-Whitney U |
|  | Erythrocyte Sedimentation Rate (First) | | 63.00 [30.00, 97.00] | 55.00 [25.00, 91.00] | .11 | Mann-Whitney U |
|  | Glucose (Blood Gas) (Last) | | 131.00 [107.00, 163.00] | 133.00 [112.00, 161.00] | .011 | Mann-Whitney U |
|  | Lactate (Last) | | 1.60 [1.10, 2.30] | 1.60 [1.10, 2.30] | .121 | Mann-Whitney U |
|  | Potassium (Blood Gas) (Last) | | 3.90 [3.50, 4.40] | 4.10 [3.70, 4.60] | <.001 | Mann-Whitney U |
|  | Albumin (Last) | | 2.80 [2.30, 3.20] | 3.10 [2.70, 3.50] | <.001 | Mann-Whitney U |
|  | Protein (Chemistry) (Last) | | 77.90 [27.25, 142.90] | 59.00 [14.70, 130.70] | <.001 | Mann-Whitney U |
|  | Cholesterol (Last) | | 116.50 [85.00, 152.00] | 150.00 [119.00, 185.00] | <.001 | Mann-Whitney U |
|  | Creatinine (Last) | | 0.90 [0.60, 1.50] | 0.90 [0.70, 1.20] | <.001 | Mann-Whitney U |
|  | Glucose (Chemistry) (Last) | | 117.00 [97.00, 147.00] | 115.00 [98.00, 142.00] | .12 | Mann-Whitney U |
|  | Magnesium (Last) | | 2.00 [1.80, 2.20] | 2.00 [1.90, 2.20] | <.001 | Mann-Whitney U |
|  | Phosphate (Last) | | 3.40 [2.70, 4.20] | 3.30 [2.70, 3.90] | <.001 | Mann-Whitney U |
|  | Potassium (Chemistry) (Last) | | 4.00 [3.70, 4.40] | 4.10 [3.80, 4.40] | .008 | Mann-Whitney U |
|  | Total Protein (Chemistry) (Last) | | 5.40 [4.80, 6.00] | 5.80 [5.10, 6.40] | <.001 | Mann-Whitney U |
|  | Transferrin (Last) | | 129.00 [100.00, 168.00] | 162.00 [128.00, 203.00] | <.001 | Mann-Whitney U |
|  | Triglycerides (Last) | | 130.00 [87.00, 197.75] | 123.00 [85.00, 192.00] | .053 | Mann-Whitney U |
|  | BUN (Last) | | 22.00 [13.00, 38.00] | 18.00 [12.00, 28.00] | <.001 | Mann-Whitney U |
|  | Lymphocytes (Absolute) (Last) | | 0.97 [0.57, 1.50] | 1.38 [0.85, 2.06] | <.001 | Mann-Whitney U |
|  | Lymphocytes (%) (Last) | | 9.00 [5.10, 15.00] | 12.00 [7.10, 18.60] | <.001 | Mann-Whitney U |
|  | Erythrocyte Sedimentation Rate (Last) | | 63.00 [33.00, 98.00] | 55.00 [25.00, 90.00] | .074 | Mann-Whitney U |
|  | Glucose (Blood Gas) (Min) | | 107.00 [87.00, 133.00] | 108.00 [92.00, 129.00] | .015 | Mann-Whitney U |
|  | Lactate (Min) | | 1.10 [0.80, 1.50] | 1.20 [0.90, 1.70] | <.001 | Mann-Whitney U |
|  | Potassium (Blood Gas) (Min) | | 3.50 [3.20, 4.00] | 3.70 [3.40, 4.10] | <.001 | Mann-Whitney U |
|  | Protein (Chemistry) (Min) | | 56.70 [15.15, 118.45] | 46.70 [11.20, 106.70] | .005 | Mann-Whitney U |
|  | Cholesterol (Min) | | 115.00 [82.25, 150.00] | 150.00 [118.00, 185.00] | <.001 | Mann-Whitney U |
|  | Magnesium (Min) | | 1.60 [1.50, 1.80] | 1.80 [1.60, 2.00] | <.001 | Mann-Whitney U |
|  | Total Protein (Chemistry) (Min) | | 5.40 [4.70, 6.00] | 5.70 [5.10, 6.40] | <.001 | Mann-Whitney U |
|  | Transferrin (Min) | | 126.00 [96.00, 164.00] | 160.00 [127.00, 201.00] | <.001 | Mann-Whitney U |
|  | Triglycerides (Min) | | 114.00 [79.00, 168.00] | 118.00 [83.00, 177.00] | .004 | Mann-Whitney U |
|  | BUN (Min) | | 12.00 [7.00, 21.00] | 13.00 [9.00, 19.00] | <.001 | Mann-Whitney U |
|  | Lymphocytes (%) (Min) | | 6.00 [3.00, 10.60] | 10.20 [5.60, 16.80] | <.001 | Mann-Whitney U |
|  | Erythrocyte Sedimentation Rate (Min) | | 60.00 [30.00, 87.00] | 52.00 [22.00, 89.00] | .322 | Mann-Whitney U |
|  | Glucose (Blood Gas) (Max) | | 162.00 [126.00, 218.50] | 167.00 [140.00, 202.00] | .009 | Mann-Whitney U |
|  | Lactate (Max) | | 2.50 [1.60, 4.00] | 2.20 [1.50, 3.40] | <.001 | Mann-Whitney U |
|  | Potassium (Blood Gas) (Max) | | 4.40 [3.80, 5.10] | 4.70 [4.10, 5.30] | <.001 | Mann-Whitney U |
|  | Albumin (Max) | | 3.00 [2.60, 3.50] | 3.30 [2.90, 3.70] | <.001 | Mann-Whitney U |
|  | Protein (Chemistry) (Max) | | 105.70 [43.40, 188.90] | 76.20 [19.00, 165.90] | <.001 | Mann-Whitney U |
|  | Cholesterol (Max) | | 119.00 [86.25, 153.75] | 151.00 [120.00, 185.00] | <.001 | Mann-Whitney U |
|  | Creatinine (Max) | | 1.30 [0.80, 2.60] | 1.10 [0.80, 1.60] | <.001 | Mann-Whitney U |
|  | Glucose (Chemistry) (Max) | | 191.00 [149.00, 277.00] | 157.00 [130.00, 209.00] | <.001 | Mann-Whitney U |
|  | Total Protein (Chemistry) (Max) | | 5.50 [4.80, 6.10] | 5.80 [5.20, 6.40] | <.001 | Mann-Whitney U |
|  | Transferrin (Max) | | 132.00 [103.00, 171.00] | 164.00 [130.00, 205.00] | <.001 | Mann-Whitney U |
|  | Triglycerides (Max) | | 147.50 [93.25, 241.00] | 128.00 [87.00, 211.00] | <.001 | Mann-Whitney U |
|  | Lymphocytes (Absolute) (Max) | | 1.31 [0.80, 2.05] | 1.57 [1.02, 2.27] | <.001 | Mann-Whitney U |
|  | Lymphocytes (%) (Max) | | 12.70 [7.60, 20.50] | 14.00 [8.90, 20.90] | <.001 | Mann-Whitney U |
|  | Erythrocyte Sedimentation Rate (Max) | | 75.00 [33.00, 110.00] | 56.00 [25.00, 93.00] | .012 | Mann-Whitney U |
