# Supplementary material for: Development and Interpretability Analysis of a Stacking Ensemble Model for Early Prediction of Nutritional Risk in Intensive Care Unit Patients: Retrospective Cohort Study
Source: JMIR Med Inform. 2026 Jun 3;14:e77872. doi: 10.2196/77872 (PMC13232782; doi:10.2196/77872)

Multimedia Appendix 5. Comprehensive Model Performance Analysis

This appendix provides a detailed visual assessment of the predictive performance for the final E-NUTRIC stacking ensemble relative to all individual base learners (XGBoost, LightGBM, Random Forest, Logistic Regression) and the clinical baseline mNUTRIC score. While the main manuscript focuses on the comparison with the standard clinical score, Figure S1 presents the Receiver Operating Characteristic (ROC) curves for all developed models. The E-NUTRIC model (purple line) demonstrates the highest overall discriminative ability, effectively enveloping the curves of the base learners and confirming that the stacking strategy successfully integrates the strengths of its components. Furthermore, Figure S2 illustrates the Precision-Recall Curves (PRC), which offer a more rigorous evaluation given the dataset’s imbalance. The E-NUTRIC model achieves a dominant position with the highest Area Under the Precision-Recall Curve, maintaining superior precision across varying recall thresholds compared to the best-performing single model and showing a substantial improvement over the mNUTRIC baseline. These curves validate the model’s robustness in minimizing false positives while accurately identifying patients at risk of malnutrition.

Figure S1. ROC curves for all models.


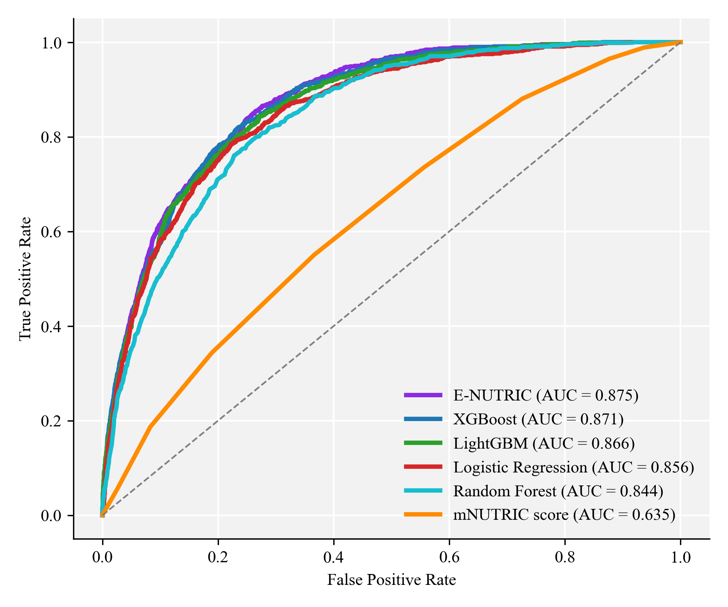


Figure S2. PRC for all models.


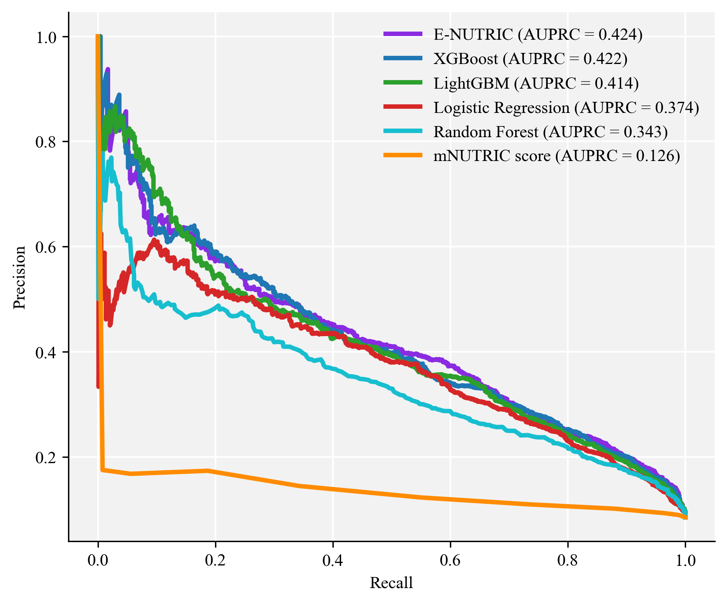

Supplement: Multimedia Appendix 5 [file medinform-v14-e77872-s005.docx]
