# Supplementary material for: Development and Interpretability Analysis of a Stacking Ensemble Model for Early Prediction of Nutritional Risk in Intensive Care Unit Patients: Retrospective Cohort Study
Source: JMIR Med Inform. 2026 Jun 3;14:e77872. doi: 10.2196/77872 (PMC13232782; doi:10.2196/77872)

Multimedia Appendix 6. SHAP Global Feature Interaction Network Plot

Figure S1. SHAP global feature interaction network plot derived from the XGBoost component. This circular network graph illustrates the multidimensional interdependencies among the top predictive features. The nodes represent individual features, with node size and color gradient (green) reflecting their global importance magnitude (Vimp). The edges (connecting lines) represent the strength of SHAP interaction values (Vint) between feature pairs. The thickness and color intensity (red) of the edges denote the interaction intensity. Notably, the strongest interaction is observed between Weight and Gender: F, indicating that the predictive risk threshold for admission weight is heavily modulated by the patient’s biological sex—a nuanced, non-linear relationship successfully captured by the tree-based ensemble.


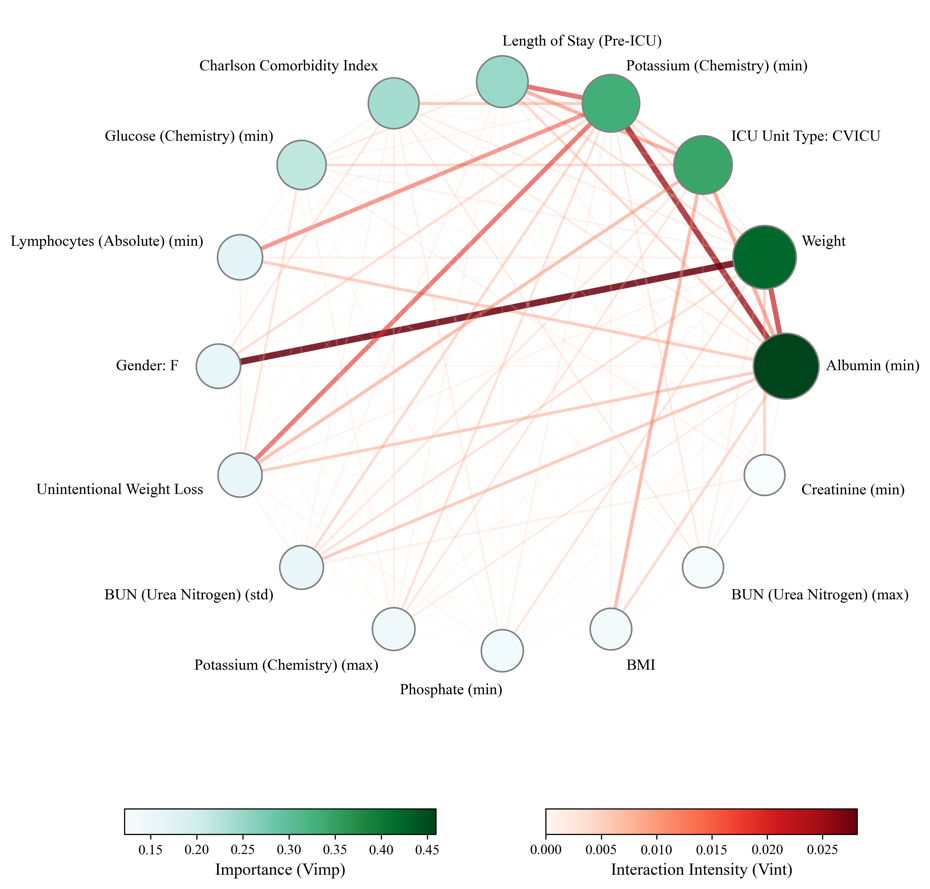

Supplement: Multimedia Appendix 6 [file medinform-v14-e77872-s006.docx]
